# Natural products’ antiangiogenic roles in gynecological cancer

**DOI:** 10.3389/fphar.2024.1353056

**Published:** 2024-05-01

**Authors:** Shangmei Jia, Ling Li, Chenghao Yu, Fu Peng

**Affiliations:** ^1^ State Key Laboratory of Southwestern Chinese Medicine Resources, Department of Basic Medicine, Chengdu University of Traditional Chinese Medicine, Chengdu, China; ^2^ West China School of Pharmacy, Sichuan University, Chengdu, China

**Keywords:** natural products, antiangiogenic, gynecological cancer, pure compound, smedicinal plants and extracts, review

## Abstract

Gynecological cancers pose a significant threat to women’s health. Although the pathogenesis of gynecological cancer remains incompletely understood, angiogenesis is widely acknowledged as a fundamental pathological mechanism driving tumor cell growth, invasion, and metastasis. Targeting angiogenesis through natural products has emerged as a crucial strategy for treating gynecological cancer. In this review, we conducted comprehensive searches in PubMed, Embase, Web of Science, Science Direct, and CNKI databases from the first publication until May 2023 to identify natural products that target angiogenesis in gynecologic tumors. Our findings revealed 63 natural products with anti-angiogenic activity against gynecological cancer. These results underscore the significance of these natural products in augmenting their anticancer effects by modulating other factors within the tumor microenvironment via their impact on angiogenesis. This article focuses on exploring the potential of natural products in targeting blood vessels within gynecological cancer to provide novel research perspectives for targeted vascular therapy while laying a solid theoretical foundation for new drug development.

## 1 Introduction

**FIGURE 1:**
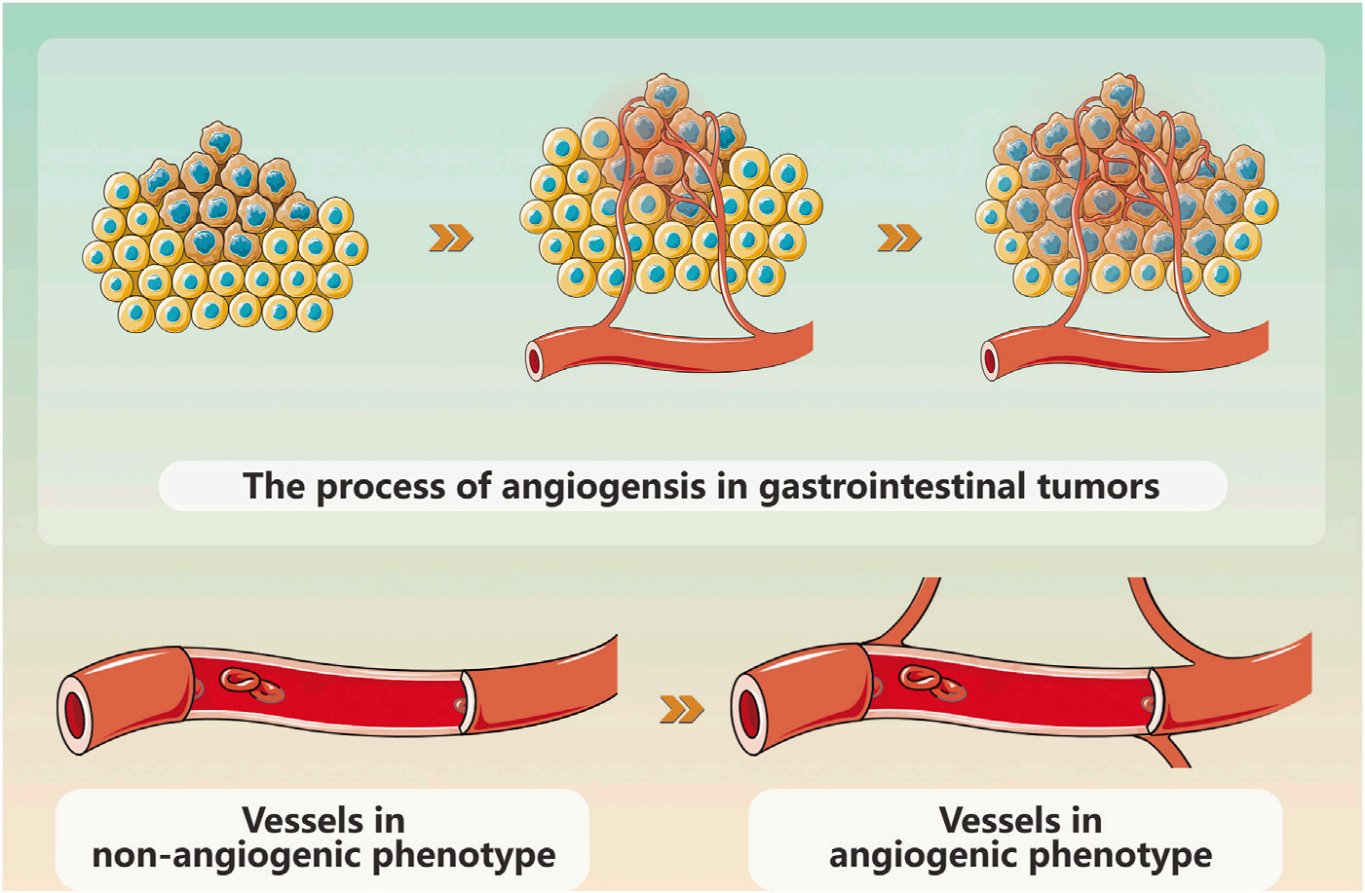
Schematic diagram of gynecological cancers angiogenesis.

**FIGURE 2:**
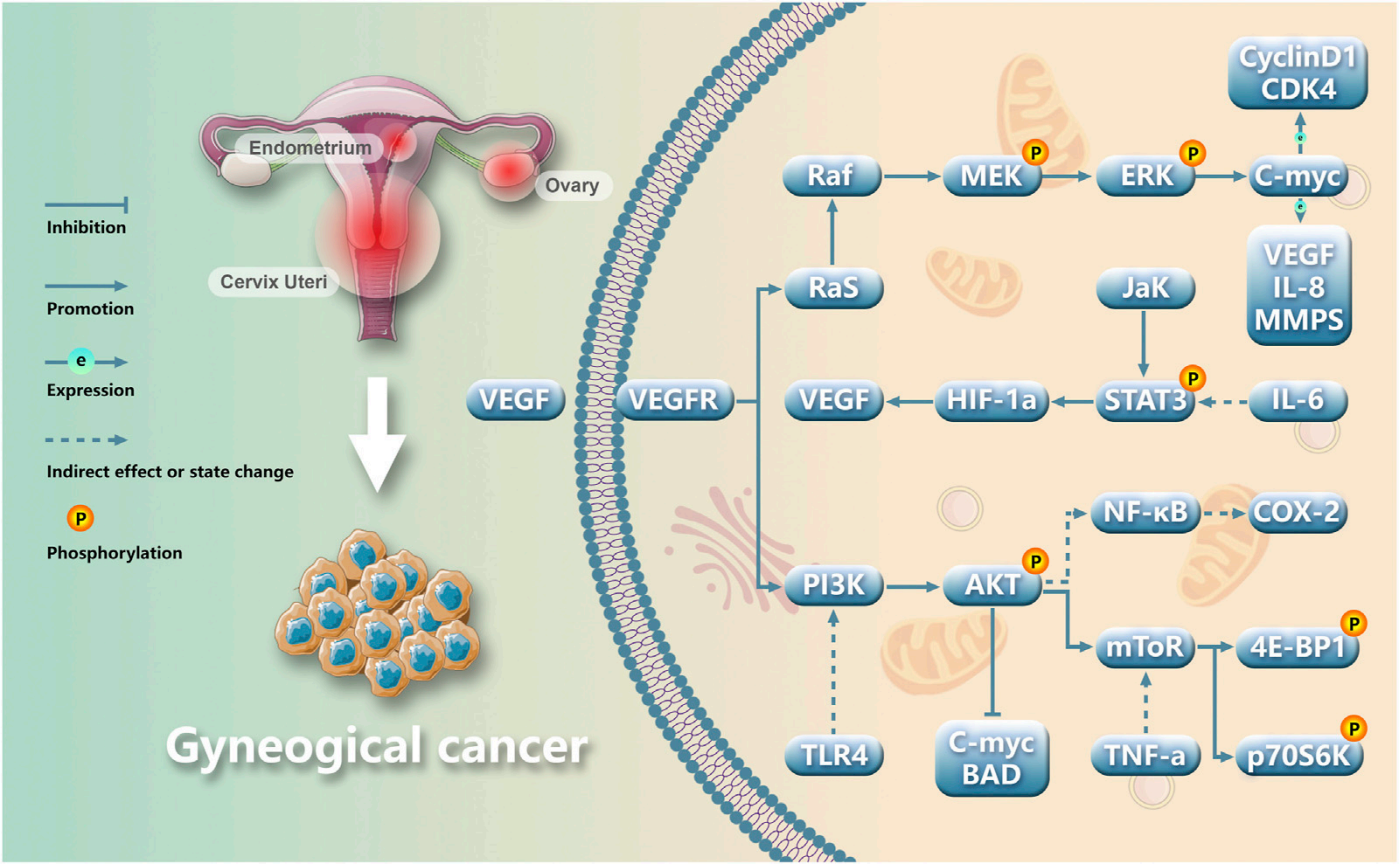
Main molecular pathways involved in gynecological cancers angiogenesis.

Gynecological cancer encompass a range of tumors, including cervical cancer, endometrial cancer, ovarian cancer, vulvar cancer, uterine sarcoma, and choriocarcinoma. Among these neoplasms, endometrial cancer (EC), cervical cancer (CC), and ovarian cancer (OC) stand out as prevalent malignant conditions in the field of gynecology. Their incidence is progressively increasing worldwide each year. Currently, modern medical treatment for gynecological cancer primarily encompasses surgery, chemoradiotherapy, hormone therapy, etc., but these approaches are associated with numerous side effects and a poor prognosis (Xiao et al., 2023). Gynecological cancer pose a threat to women’s health and their occurrence and progression rely on the provision of blood resulting in the formation of multiple tumor neovascularizations (Xiao et al., 2020) (Figure 1). Angiogenesis is crucial for maintaining homeostasis and plays a pivotal role in tumor proliferation, advancement, and metastasis. The imbalance between pro-angiogenic factors and anti-angiogenic factors leads to pathological angiogenesis; therefore, inhibiting angiogenesis represents an effective approach to impede tumor growth (Figure 2). Vascular endothelial growth factor (VEGF) serves as a key growth factor that stimulates angiogenesis while targeted anti-angiogenic drugs have demonstrated significant efficacy in the clinical management of various tumors. VEGF participates in diverse stages of gynecological cancer development by playing a central role in establishing and sustaining the tumor vascular system.

A variety of natural products, such as paclitaxel, compbrestatine, and camptothecin, are considered to be potent angiogenesis inhibitors with minimal toxicity. Natural products have always played a crucial role in the discovery of new drugs. From 1999 to 2013, approximately 28% of top-tier drugs approved by the U.S. Food and Drug Administration (FDA) were derived from natural pharmacophores. Currently, around 70% of anti-cancer drugs available on the market have been developed from natural products and their derivatives (Ding et al., 2016). Such as resveratrol, curcumin, and epigallocatechin-3-gallate (EGCG) have demonstrated significant chemotherapeutic activity against various types of cancer (Delmas et al., 2003; Dhillon et al., 2008).

Compared to chemically synthesized drugs, natural products possess unique advantages in terms of structural diversification, multi-component composition, multi-target activity, and superior efficacy compared to certain drugs targeting angiogenesis factors. Simultaneously, natural products exhibit minimal side effects and excellent tolerance, making them a crucial area for new drug development. This paper presents the first study and review on the regulation of natural products on angiogenesis in gynecological cancer. The aim is to provide theoretical, scientific, and clinical evidence supporting the use of natural products in the prevention and treatment of gynecological cancer.

## 2 Methods

We conducted a comprehensive search of PubMed, Embase, Web of Science, ScienceDirect, and CNKI from the original publication date to May 2023 to investigate the mechanisms through which natural products exert their anti-angiogenic potential in inhibiting the progression of gynecological cancer. The literature included in this review encompasses three main categories:

(1) “gynecological cancer” and its synonyms, including “gynecological tumor,” “gynecological malignancy,” “cervical cancer,” “ovarian cancer,” “endometrial cancer,” “vulvar cancer,” “uterine sarcoma,” and “choriocarcinoma.” (2) “anti-angiogenesis” and “vascular endothelial growth factors” including “tumor angiogenesis” and “VEGF.” (3) “natural products” and related terms like “natural compounds” and “herbs.” An initial database search yielded 6,549 records, which were then screened for duplicates resulting in 3,683 unique studies. After evaluating these studies based on title and abstract, we excluded 3,620 articles leaving us with a final selection of 63 relevant publications. It is worth noting that most of the included studies are either *in vivo* or *in vitro* experiments or preclinical investigations; however, there is a scarcity of human studies.

## 3 Results

### 3.1 Natural compounds targeting angiogenesis in gynecological cancer

**FIGURE 3:**
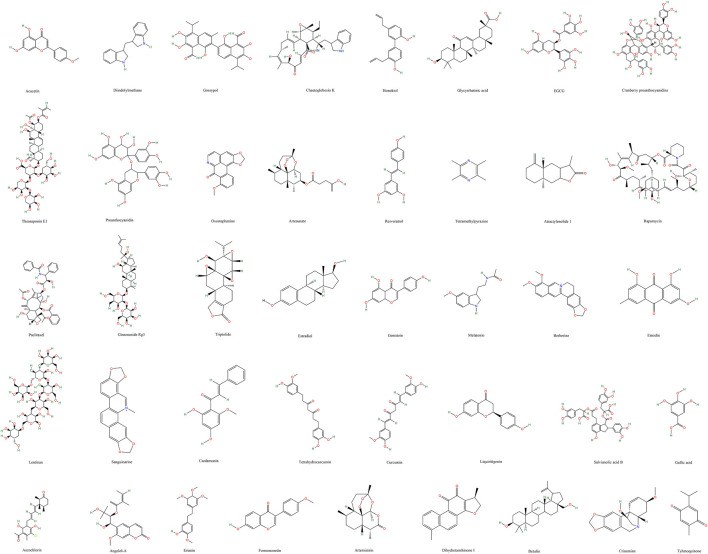
Chemical structures of natural compounds targeting gynecological cancer angiogenesis.

(Supplementary Table S1; Figure 3)

#### 3.1.1 Terpenoids

Terpenoids are essential constituents of natural organic compounds, with over 50,000 reported varieties. Natural terpenoids exhibit potent anti-tumor, anti-inflammatory, and antibacterial activities. These include sesquiterpenoids, diterpenoids, monoterpenoids, disesquiterpenoids, and triterpenoids that contain one or more carbon atoms with double or triple bonds. They are widely distributed in plants, microorganisms, marine organisms, and certain insects (Liyuan et al., 2024).

The artemisinin, derived from the Chinese herb *Artemisia caruifolia* Buch.-Ham. ex Roxb. (Ma et al., 2020). Some studies have demonstrated that the expression of relevant genes and proteins was assessed using RT-qPCR and Western blot analysis with a concentration of 300 μM artemisinin. The findings revealed a significant downregulation of ERa and VEGF expression by artemisinin, thereby further confirming its potential anti-angiogenic effect in cervical cancer cells. Moreover, artemisinin reduced telomerase activity, hTR and hTERT subunits, as well as the expression of HPV-39 virus E6 and E7 components, while increasing p53 expression. These results provide additional evidence supporting the reliance on p53 for artemisinin-induced apoptosis. Overall, these findings suggest that artemisinin exerts anti-proliferative and pro-apoptotic effects in HPV-39-infected ME-180 cells. However, the lack of an *in vivo* experiment and absence of a positive control group in this study preclude confirmation of artemisinin’s superiority (Teodoro et al., 2007; Mondal and Chatterji, 2015).

The semi-synthetic derivative of artemisinin, known as artesunate (ART), exhibits superior efficacy as an antimalarial drug compared to artemisinin (Moore et al., 1995; Efferth et al., 2001; Wu et al., 2001; Efferth et al., 2002). Through *in vivo* and *in vitro* studies, ART shows promise as an effective inhibitor of angiogenesis. Human ovarian cancer HO-8910 cells were implanted into nude mice, and four groups were established: a control group and ART high, medium, and low dose groups (10, 50, and 100 mg/kg). Subcutaneous injections of ART were administered daily. After 15 days of treatment, the tumors were removed and immunohistochemistry was performed to detect CD31 expression as well as VEGF levels and VEGF receptor KDR/flk-1 expression. It was observed that the expression of these markers decreased following drug treatment, resulting in slowed tumor growth and reduced microvascular density. Furthermore, no evident toxicity to animals was observed. The angiogenesis model using HUVEC cells was also tested. The results demonstrated that ART significantly suppressed VEGF expression on HO-8910 cells as well as KDR/flk-1 expression on both HUVEC cells and HO-8910 cells. Moreover, ART exhibited a dose-dependent inhibition of angiogenesis within the range of 0.5–50 μmol/L (Chen et al., 2004).

Betulin, an essential compound derived from the bark of *Betula pendula subsp. mandshurica* (Regel) Ashburner & McAll., can be converted into betulinic acid. Dehelean et al. (2012) investigated the cytotoxic effects of betulin (3, 10, 30 μM) on HeLa, MCF7, and A431 cancer cells and elucidated the underlying apoptotic mechanism. Additionally, they explored the correlation between betulin and angiogenesis in the chorioallantoic membrane of chicken embryos. The results demonstrated that betulin exhibited a superior inhibitory effect on cervical cancer cells. At higher concentrations, betulin induced cell membrane disruption, thereby sustaining its apoptotic impact. Betulin prompted a reduction in neovascularization and appeared to function as a drug targeting vascular endothelial cells, potentially through apoptosis-mediated mechanisms, leading to diminished functional blood vessels within the rapidly proliferating capillary cluster in the chorio-allantoic membrane of chicken embryos. These findings underscored the significant dose-dependent anti-tumor and anti-angiogenic activities of betulin. However, there are certain limitations in the experimental design such as the absence of a positive control group and limited relevant detection indicators; thus further verification is required to ensure the reliability of these experimental outcomes.

Ginsenoside Rg3, a monomeric compound extracted from *Panax ginseng* C.A.Mey., is a trace element in traditional Chinese medicine that has been shown to inhibit the adhesion, invasion, and proliferation of tumor cells as well as the formation of tumor neovascularization. The cervical cancer-bearing nude mouse model was treated with ginsenoside Rg3 (5 mg/kg) via oral gavage for a duration of 5 weeks. Both a cisplatin positive control group and a combined treatment group were established. Immunohistochemical analysis was conducted to determine the expression of CD31 and PCNA in tumor tissues, while apoptosis was assessed using the TUNEL method. The results demonstrated that ginsenoside Rg3, either alone or in combination with cisplatin, effectively inhibited the growth of Hela cell transplanted tumors in nude mice. Furthermore, it downregulated the expression of CD31 and PCNA, thereby inhibiting angiogenesis in cervical cancer. Additionally, it enhanced the drug sensitivity of cisplatin towards cervical cancer cells and mitigated its associated side effects (Yingchun, 2007) Moreover, a study established an abdominal cavity transplantation tumor model of severe combined immunodeficiency (SCID) mice with ovarian cancer and divided them into three groups: blank group, control group, and experimental group (ginsenoside Rg3, 0.75 mg/mL), which underwent continuous gavage treatment for 26 days. VEGF mRNA, protein expression, and microvascular density (MVD) were assessed through reverse transcription polymerase chain reaction, enzyme-linked immunosorbent assay, and immunohistochemistry respectively. The findings revealed that ginsenoside Rg3 suppressed tumor growth and metastasis by down-regulating VEGF mRNA and protein expression (Zimin et al., 2002). However, pharmacological toxicity or clinical-related experimental studies were not conducted in these two experiments; hence the safety profile and clinical therapeutic effect require further investigation.

Additionally, we have identified a variety of terpenoid compounds (Supplementary Table S1), which also play a significant role in the treatment of gynecological cancers.

#### 3.1.2 Quinones

Anthraquinone compounds are naturally present in various plant families, including polygonaceae, rhamnaceae, rubiaceae, legumes, scrophulariaceae, liliaceae. They can also be found in small amounts in lichens, fungi and animal metabolites. These compounds exhibit antibacterial, anti-inflammatory, antiviral and antioxidant effects. Moreover, they have the ability to influence tumor cell proliferation and differentiation as well as induce tumor cell decay. Additionally, they can reverse multidrug resistance (NMR) associated with malignant tumors. Natural anthraquinones come in a variety of forms with distinct structures and pharmacological activities (Mingming et al., 2019).

The impact of emodin on the proliferation of U14 cervical cancer cells transplanted in mice was investigated by several scholars (Zhang et al., 2015). The mice were divided into four groups: a control group (treated with DMSO), a low-dose emodin group (20 mg/kg), a high-dose emodin group (40 mg/kg), and a cisplatin group (3 mg/kg). They received oral administration for 26 days. MVD was assessed using immunohistochemistry. Real-time quantitative RT-qPCR and Western blot were employed to analyze the mRNA and protein levels of HIF-1α, VEGF, and MIF, respectively. Results showed that CD34, HIF-1α, VEGF, MIF, and Bcl-2 levels significantly decreased while Bax levels significantly increased following treatment with emodin. These findings suggest that emodin may inhibit cervical cancer in mice by suppressing tumor neovascularization, reducing MIF expression, and promoting tumor cell apoptosis. The experiment solely focused on *in vivo* studies, without conducting any *in vitro* experimental studies to enhance the experimental design and validate the data’s reliability.

The bioactive compound dihydrotanshinone I (DHT) is present in *Salvia miltiorrhiza* Bunge. DHT exhibits the ability to deactivate the NF-κB signaling pathway, thereby suppressing the expression of COX-2, MMP-9, and VEGF. Consequently, it effectively inhibits tumor cell invasion and angiogenesis (Wang et al., 2015). The *in vitro* experiments demonstrated that DHT effectively dose-dependently inhibited the expression of the NF-κB reporter gene induced by TNF-α. Moreover, it inhibited the phosphorylation and degradation of IκBα, as well as the phosphorylation and nuclear translocation of p65. Additionally, DHT downregulated the expression levels of cIAP-1, FLIP, COX-2, MMP-9, VEGF, TNF-α, IL-6 and MCP1. Furthermore, DHT significantly impeded the activation of ERK1/2, p38 and JNK/SAPK. The *in vivo* studies have demonstrated that DHT effectively suppresses the growth of HeLa cells in xenograft tumor models, potentially through its regulation of TNF-α production. The summary indicates that DHT has demonstrated the ability to inhibit NF-κB and its regulatory gene products, which are associated with various beneficial effects including anti-proliferative, pro-apoptotic, anti-invasive, anti-angiogenic, and anti-inflammatory properties (Wang et al., 2015). The lack of a positive control group in this experiment unfortunately undermines its credibility to some extent.

The anti-proliferative effects of *Nigella sativa* L. (NS) seed ethanol extract and its volatile oil thymoquinone (THM) on SKOV3 cells were investigated at varying drug concentrations (4μM, 4μM, 100μM, and 100 μM), with the drug concentration adjusted to NS (50 μg)/THM (50 μM) based on IC50. After a 24-h intervention in SKOV3 cells, Western blot was used to detect the expression levels of related proteins. Results in comparison to the control group, docetaxel (DTX), paclitaxel (PTX), NS, THM, and DTX/PTX + NS/THM significantly induced elevated levels of IL-8 and pro-apoptotic protein Bax. Conversely, the expression of TNF-α, VEGF, and Bcl-2 protein was downregulated. The anti-proliferative, anti-angiogenic, and pro-apoptotic activities exhibited by NS and THM clearly demonstrate their potential in combating ovarian cancer. NS and THM alone or in combination with DTX and PTX inhibit SKOV3 growth through pathways mediated by IL-8, TNF-α, VEGF as well as Bax/Bcl-2 (Mathur and Tiwari, 2016). Although the study confirmed the anticancer effects of NS and THM in ovarian cancer, further verification is necessary due to the lack of *in vivo* experiments and unclear identification of therapeutic components within the NS ethanol extract.

#### 3.1.3 Glycosides

Glycosides are natural products synthesized from various plants, possessing pharmacological properties such as anti-inflammatory, anti-tumor, anti-oxidative, anti-fibrotic, anti-allergic, antidiabetic, antidepressant and antifungal effects. Additionally, they enhance the body’s immunity and have cardioprotective and neuroprotective effects by dilating blood vessels and preventing arrhythmia (Xingwen et al., 2023).

The securidaca saponin was isolated from a purified root extract of *Securidaca longepedunculata* Fresen. Previous studies have identified the main components extracted from the plant (4A3 and 4A4) as triterpenoid glycosides (Obasi et al., 2018). Treatment with 4A3 and 4A4 at concentrations of 7.03 and 16.39 μg/mL respectively significantly attenuated cell migration in Caski and Bu25 TK cells after both 48 h and 72 h of exposure. The RT-qPCR analysis revealed that the expression of MCL-1, BCL2L1, AKT-3, VEGFA, and MALAT1 was downregulated through modulation of the PI3K-AKT/mTOR/NF-κB signaling pathway. The findings demonstrated that 4A3 and 4A4 effectively suppressed cancer cell proliferation, induced apoptosis, inhibited angiogenesis, and hindered cervical cancer cell survival through diverse mechanisms (Obasi et al., 2018). The experiment, however, also revealed certain limitations as it did not include *in vivo* experimentation and lacked a positive control group. Therefore, further verification is required to ensure the reliability of the experimental data.

Teasaponin E_1_ (TSE_1_) is an oleanane-type saponin derived from the seeds of *Camellia sinensis* (L.) Kuntze. Li et al. (2021) conducted *in vitro* experiments and chicken chorio-allantoic membrane (CAM) experiments to investigate the apoptosis-inducing, cell cycle arrest, and anti-angiogenesis activities of TSE_1_ on platinum-resistant ovarian cancer cells. The results demonstrated that TSE_1_ effectively suppressed the growth of ovarian OVCAR-3 and A2780/CP70 cells in a concentration-dependent manner while exhibiting minimal toxicity towards normal ovarian IOSE-364 cells. In this experiment, They established the Control group, TSE_1_ group (2 µM), Akt signaling inhibitor (wortmannin) group (100 nM), Notch1 signaling inhibitor (DAPT) group (80 µM), as well as the TSE_1_+ DAPT/wortmannin groups. The results demonstrated that TSE_1_ effectively induced apoptosis in OVCAR-3 cells through both endogenous and exogenous apoptotic pathways. Additionally, it modestly arrested the cell cycle at the G2/M phase and significantly suppressed OVCAR-3 cell proliferation by downregulating the protein expression of VEGF, HIF-1α, 4E-BP1, NICD, Dll4, and Jagged1. Moreover, TSE_1_ exhibited inhibitory effects on migration and angiogenesis. To evaluate its antiangiogenic activity *in vivo*, we employed the CAM assay. Compared to the control group (TSE_1_ with a drug concentration of 4 µM), TSE_1_ remarkably reduced both the number and density of CAM vessels. In summary, the TSE_1_ may potentially enhance the treatment of platinum-resistant ovarian cancer through modulation of the Dll4/Jagged1-Notch1-Akt-HIF-1α signaling pathway. However, the absence of a drug-positive control group in this experiment, compromised the study’s reliability. Additionally, another experimental study demonstrated the potent anti-proliferative effects of TSE_1_ on OVCAR-3 and A2780/CP70 ovarian cancer cells. Furthermore, TSE_1_ appears to induce apoptosis in human ovarian cancer cells via an exogenous pathway and reduce VEGF protein levels through an HIF-1α-dependent mechanism, thereby effectively inhibiting angiogenesis in these cells (Jia et al., 2017).

#### 3.1.4 Alkaloids

Alkaloids are a class of significant natural bioactive compounds in traditional Chinese medicine. They are organic nitrogen-containing compounds that occur naturally, primarily in plants with a few originating from animals. Possessing intricate ring structures and predominantly alkaline properties, they exhibit diverse functionalities including analgesic, antibacterial, anti-inflammatory effects as well as cough and asthma relief and anticancer activities (Lu et al., 2022).

Sanguinarine is widely distributed in nature and derived from *Macleaya cordata* (Willd.) R. Br. Its primary constituents belong to the isoquinoline alkaloids. Through *in vitro* cell experiments, Jiaying (2013). discovered that sanguinarine exhibits concentration- and time-dependent inhibition on the adhesion, migration, movement, invasion, and metastasis of HeLa and Siha cells derived from cervical cancer. Moreover, it significantly suppresses cell invasion and metastasis at non-toxic doses. The underlying mechanism involves upregulation of E-cadherin and PTEN protein expression levels while downregulating α-catenin, β-catenin, γ-catenin, MMP-2, MMP-9, and VEGF expression levels. In the *in vitro* study, a total of 40 nude mice were divided into four groups: normal saline control group, cisplatin positive control group (5 mg/kg), sanguinarine low concentration group (1.25 mg/kg), and sanguinarine high concentration group (2.5 mg/kg). The mice were treated for a duration of 28 days. Immunohistochemistry, Western blot analysis, and transmission electron microscopy techniques were employed to evaluate the effects. The findings revealed that treatment with hemagglutinine resulted in downregulation of CD34, Ki67, EGFR, Bcl-2, and VEGF expressions in tumor tissues while up-regulating the pro-apoptotic protein Bax expression. These results indicate that sanguinarine possesses inhibitory properties against xenografted cervical cancer growth *in vivo* by exerting anti-angiogenic effects, suppressing tumor cell proliferation, inducing apoptosis and autophagy mechanisms. Although the experimental design was relatively comprehensive, one limitation was the absence of a positive control group in the cell experiment.

Crinamine is primarily derived from the bulbs of *Crinum asiaticum* L. Khumkhrong et al. (2019) demonstrated, for the first time, the mechanism of action of crinamine in inhibiting cervical cancer. It exhibits superior inhibition of unanchored tumor sphere growth compared to existing chemotherapy agents carboplatin and 5-fluorouracil or CDK9 inhibitor FIT-039. Crinamine induces cell apoptosis without promoting DNA double strand breaks and suppresses cervical cancer cell migration by downregulating SNAI1 and VIM expression, positive regulators of epithelial-mesenchymal transition. Notably, crinamine also exerts anti-angiogenic activity by inhibiting VEGFA protein secretion and vascular development in zebrafish embryonic cervical cancer cells. Gene expression analysis suggests that its mechanism of action may involve downregulation of cancer-related genes such as AKT1, BCL2L1, CCND1, CDK4, PLK1, and RHOA.

The study conducted by Tran et al. (2022). extracted oxostephanine from the leaves of *Stephania dielsiana* Y.C.Wu., and demonstrated its potential as an aurora kinase inhibitor. A comparison was made between oxostephine and the aurora kinase inhibitor VX-680 in terms of their effects on OVCAR-8 cell growth in ovarian cancer. The results revealed that oxostephine effectively inhibited the growth and migration of OVCAR-8 cells, while selectively exhibiting cytotoxicity towards HUVEC but demonstrating lower toxicity towards hFBs cells. Furthermore, oxostephine downregulated the expression of VEGFA, HGF, and FG2 in both HUVEC and hFBs cells, indicating its role in regulating cell proliferation, migration, and angiogenesis. In conclusion, a limited number of aurora kinase inhibitors have been identified and developed for clinical trials in cancer treatment with some degree of efficacy observed. However, further testing is required to evaluate the impact of oxostephine on other ovarian cancer cell lines, particularly primary cell lines to validate its effectiveness against ovarian cancer. Moreover, it is crucial to conduct *in vivo* tumor model experiments to confirm the inhibitory effects of oxostephine on both auroral kinase activity and angiogenesis.

#### 3.1.5 Polyphenols

Polyphenol compounds are secondary metabolites synthesized by plant tissues, characterized by the presence of multiple phenolic groups. They are widely distributed in various plant-based foods and can be derived from a variety of fruits, vegetables, tea, coffee, wine, and whole wheat products (Fengxia and Zeli, 2023; Yaru and Wentao, 2023).

The phenolic compound curcumin (CUR) extracted from *Curcuma longa* L. has been demonstrated in numerous recent studies to exert regulatory effects on angiogenesis, proliferation, invasion, and tumor progression across various cancer types. Yoysungnoen-Chintana et al. (2014) discovered thatm CUR effectively inhibited tumor progression and angiogenesis in nude mice transplanted with cervical cancer cells (CaSki-). A total of 36 nude mice were divided into six groups: Control + vehicle (corn oil); Control + CUR (1,500 mg/kg); CaSki + vehicle (corn oil); CaSki + CUR (500 mg/kg); CaSki + CUR (1,000 mg/kg); CaSki + CUR (1,500 mg/kg) for a duration of 30 days. The immunohistochemical analysis revealed a significant reduction in the expression levels of CD31, VEGF, COX-2, and EGFR. In conclusion, the high-dose administration of CUR demonstrated its potential to suppress tumor growth and angiogenesis in caski-transplanted mice through the downregulation of VEGF, COX-2, and EGFR signaling pathways. Although this experiment confirmed the anti-cervical cancer effect of CUR, the absence of a positive control group and the omission of any experimental study on pharmacological toxicity raise concerns regarding safety and call for further verification of the reliability of experimental data.

Tetrahydrocurcumin (THC) is a significant metabolite of CUR *in vivo*. Research has demonstrated that THC exhibits more potent anti-angiogenesis activity against tumors compared to CUR (Yoysungnoen et al., 2008), which may be attributed to its higher antioxidant activity. Female BALB/c nude mice were divided into a control group and a CaSki group, with daily oral administration of CUR 100, 300, and 500 mg/kg for 30 days. MVD was assessed by CD31 expression, while the expressions of VEGF, VEGFR-2 and HIF-1 were detected through immunohistochemistry. The MVD in the CaSki + loading group was significantly higher than that in the CON + loading group; however, treatment with high, medium and low doses of THC resulted in a significant reduction in MVD among the CaSki group. Furthermore, while expressions of VEGF, VEGFR-2 and HIF-1 were significantly increased in the CaSki + loading group compared to controls without intervention; these expressions were downregulated by THC intervention. Thus suggesting that THC can inhibit tumor angiogenesis in caski transplanted nude mice via downregulation of HIF-1α and VEGF as well as its receptor’s expression levels (Yoysungnoen et al., 2015). It is important to note that this experiment did not include a positive control group nor conduct experimental studies related to pharmacological toxicity; therefore further verification is necessary.

EGCG (e)-epigallocatechin-3-gallate, a predominant polyphenol found in *Camellia sinensis* (L.) Kuntze, exhibits potent anticancer properties. Numerous studies have demonstrated the potent anti-cancer and cancer prevention effects of *C. sinensis* extract and EGCG in various human cancers (Yang, 1997; Sato, 1999; Lambert and Yang, 2003). Both *C. sinensis* extract and EGCG can effectively suppress the expression of HIF-1α and VEGF in HeLa cells through the PI3K/Akt and ERK1/2 signaling pathways, thereby inhibiting angiogenesis (Zhang et al., 2006). Another study demonstrated that EGCG can attenuate the expression of VEGF induced by basal and ET-1, as well as the activity of MMPs and uPA/uPA receptor, thereby inhibiting cell invasion. These findings suggest that EGCG’s suppression of the ET-1/ETAR autocrine loop may contribute to its anti-angiogenic and anti-invasive activities in ovarian cancer (Spinella et al., 2006). The absence of a positive control group in both trials precluded the determination of the effect. However, EGCG’s clinical application is limited due to poor bioavailability and the requirement for high doses to achieve therapeutic effects. The production of pro-polyphenol (−)-epigallocatechin-3-gallate (−) through acetylation of EGCG has been found to act as a prodrug, enhancing stability and bioavailability both *in vitro* and *in vivo* (Man et al., 2020).

The role of polyphenols in the prevention of gynecological cancer is significant, as evidenced by numerous additional compounds (Supplementary Table S1) with similar properties.

#### 3.1.6 Flavonoids

Flavonoids, as one of the three major secondary metabolites in plants, play crucial roles in plant growth and development, resistance to external adverse environments, and human nutrition and health. The flavonoid components are generally classified into seven categories: flavone, flavonol, isoflavone, flavanone, flavanol, anthocyanidin, and chalcone. The compounds exhibit a range of pharmacological effects, such as anti-inflammatory properties, antiviral activities, cardioprotective effects against heart diseases, anticancer properties, and anti-aging effects (Chao et al., 2023).

The flavonoid liquiritigenin (7,4′-dihydroxyflavanone, LQ), extracted from *Glycyrrhiza glabra* L., possesses antioxidant, anti-inflammatory, and anticancer properties (Kim et al., 2009; Kim et al., 2011). The cell survival rate was significantly reduced in a dose-time dependent manner when HUVEC and HeLa cells were treated with various concentrations of LQ (25, 50, 75, 100 μM). Western blot analysis revealed that LQ effectively inhibited the expression of VEGF and HIF-1α in both cell types, exhibiting a dose-dependent effect. Subsequent treatment with specific inhibitors including LY294002 (PI3K inhibitor), Rapamycin (p70S6K inhibitor), and U0126 (ERK1/2 inhibitor) resulted in downregulation of VEGF and HIF-1α expression mediated by the PI3K/AKT/p70S6K and ERK1/2 signaling pathways. These findings suggest that the inhibitory mechanism of LQ on HeLa cells may be associated with the PI3K/AKT/mTOR-p70S6K signaling pathway (Xie et al., 2012). Furthermore, research has demonstrated that LQ can downregulate the expression of HIF-1a and VEGF in human cervical cancer cells through the VEGF signaling pathway, thereby exerting an anti-angiogenic effec (Liu et al., 2012). The absence of a positive control group and the lack of animal experimental studies in both experiments necessitate further verification to ensure the reliability of the experimental data.

Formononetin, a naturally occurring isoflavone that can be extracted from *Astragalus mongholicus* Bunge, exhibits diverse biological functions including anti-inflammatory, antioxidant, and hepatoprotective effects (Zheng et al., 2020). The inhibitory effects of onononin on RAS/ERK and JAK1/STAT3 signaling pathways have been validated through *in vitro* and *in vivo* studies, leading to the regulation of STAT3, PD-L1, and MYC-related targets. Ultimately, this mechanism is the impediment of cervical cancer progression and angiogenesis (Wang et al., 2022). The trial lacked a positive control group, thus preventing the confirmation of formononetin’s superiority.

The flavonoid compound acacetin (5,7-dihydroxy-40-methoxyflavone) is commonly present in various plants, seeds, and flowers and exhibits anticancer properties. The OVCAR-3/A2780 cells were subjected to treatment with varying concentrations of acacetin (10, 20, 30 μM). RT-qPCR and Western blot analyses revealed that acacetin downregulated the expression of HIF-1a and AKT. The number of blood vessels was assessed through CAM experimentation, while the expressions of HIF-1 and VEGF were detected using Western blot and RT-qPCR techniques. The findings revealed a decrease in blood vessel count, as well as a reduction in HIF-1a and VEGF expression upon treatment with acacetin. In conclusion, acacetin hinders ovarian cancer progression and angiogenesis by suppressing VEGF expression via inhibition of the AKT/HIF-1 signaling pathway (Liu et al., 2011). Notably, the study lacked a positive control group and did not include any animal experiments.

#### 3.1.7 Phenylpropanoids

Phenylpropanoids can be classified into various chemical categories, including simple phenylpropanols, coumarins and lignans. Coumarins is a natural product characterized by its aromatic nature and widespread occurrence in plants such as Apiaceae, Rutaceae, Asteraceae, legumes, orchids, among others. In recent years, coumarin-containing natural products have gained significant attention in clinical applications due to their remarkable therapeutic effects such as antioxidative, anti-tumoral, antibacterial, antiviral, anti-inflammatory properties as well as neuroprotective activities.

Angelol-A is a coumarin compound extracted from the root of *Angelica pubescens*. The study conducted by Ying et al. (2022), demonstrated the treatment of cervical cancer cells with varying concentrations of angelol-a (40, 80, 120 μM). Cell viability was assessed using the MTT assay, while cell cycle analysis was performed by PI staining. The expression of miR-29a-3p, *in vitro* migration/invasion, and angiogenesis were evaluated in angelol-a-treated cells with/without antago-miR-29a-3p (miR-29a-3p inhibitor) or U0126 (MEK inhibitor), using RT-qPCR for miR-29a-3p expression analysis, a chemotaxis assay for migration/invasion assessment, and a tube formation assay for angiogenesis evaluation. The expressions of MMP2, MMP9, and VEGFA were detected using Western blot analysis. It was observed that angelol-a significantly suppressed the expression of MMP2 and VEGFA, as well as inhibited cell migration and invasive behavior in human cervical cancer cells. Moreover, angelol-a exhibited inhibitory effects on invasion, motility, and angiogenesis by upregulating miR-29a-3p expression in the targeted vegf-3′utr region. These findings provide novel evidence for the anti-metastatic and anti-angiogenic properties of angelol-a in human cervical cancer cells through modulation of the ERK pathway and targeting the miR-29a-3p/MMP2/VEGFA axis.

However, the experiment had certain limitations and was not tested *in vivo*.

Honokiol, is a bioactive compound that is extracted from *Magnolia officinalis* Rehder & E.H.Wilson. and classified as a lignin compound, exhibits diverse pharmacological effects including antioxidation, anti-thrombosis, anti-inflammation, xanthine oxidase inhibition, anxiolytic properties, as well as antitumor activity. Li et al. (2008) investigated the *in vitro* and *in vivo* antitumor activity of Honokiol against human ovarian tumors. Cell proliferation and apoptosis were assessed using MTT, DNA ladder, Hoechst staining, and flow cytometry assays. The results demonstrated a dose- and time-dependent downregulation of Bcl-XL expression and upregulation of Bad expression. In the *in vivo* study, nude mice were randomly divided into three groups: Control group (PBS 100 mL), liposome group (0.6 mg/100 mL PBS), and Honokiol group (1 mg liposome-encapsulated honokiol [40%]/100 mL PBS). Intraperitoneal injections were administered daily for 56 days. The findings revealed that magnolol reduced VEGF and CD31 expression, decreased microvessel density, and inhibited tumor growth. These results suggest that Honokiol induces apoptosis and inhibits angiogenesis both *in vitro* and *in vivo*. It is worth noting that this experiment lacks a positive control group, thus further verification is required to ensure the reliability of the findings.

#### 3.1.8 Bibenzyls

The most prevalent benzyls compound in liverwort is bisbibenzyl, which exhibits significant biological activities including antifungal, antimicrobial, antioxidant, and anti-platelet aggregation properties. Bibenzyl compounds are distinctive polyphenolic aromatic compounds found in bryophytes. Their remarkable biological activities have well-defined mechanisms and targets (Juan, 2013).

Erianin, derived from *Dendrobium chrysotoxum* Lindl., is a naturally occurring dibenzyl compound with antioxidant, anti-angiogenic, anti-tumor, antibacterial, antiviral, and other pharmacological effects (Kamory et al., 1995; Manfredi et al., 2001). The antitumor activity of erianin was evaluated by Yang et al. (2021), using an *in vitro* HeLa cell model and an *in vivo* cervical cancer xenotransplantation model. In the *in vitro* experiments, various concentrations of erianin (1, 3, 10, 30, and 100 μM) were administered to HeLa cells for durations of 12, 24, or 48 h. The impact of erianin on proteins, genes, and pathways associated with its antitumor activity was investigated through MTT assays, Western blot analysis, RT-qPCR analysis, and immunoprecipitation techniques, etc. Experimental findings demonstrated that erianin suppressed PD-L1 expression while promoting its lysosomal degradation; it also downregulated the expression levels of VEGF and MMP-9. Furthermore, erianin disrupted the interaction between RAS and HIF-1α. Notably, co-culture experiments involving T-cells and HeLa cells confirmed that erianin restored cytotoxic T lymphocytes’ ability to eliminate tumor cells. In the *in vivo* experiment, nude mice were randomly divided into four groups: the erianin low-dose group (25 mg/kg), high-dose group (75 mg/kg), 5-FU group (48 mg/kg), and control group. The treatment duration was 33 days in total. Western blot analysis of tumor tissues revealed that erianin concentration-dependently reduced the protein levels of PD-L1, HIF-1α, RAS, and VEGF, exhibiting a superior inhibitory effect compared to 5-FU. Furthermore, immunohistochemical analysis demonstrated that erianin significantly decreased the expression of PD-L1, HIF-1α, RAS, and VEGF when compared with the control group. Mechanistically, erianin inhibited HIF-1α synthesis through the mTOR/p70S6K/4EBP1 pathway and suppressed RAS/Raf/MEK/MAPK-ERK signaling cascade. Immunoprecipitation experiments indicated that erianin effectively hindered PD-L1-mediated angiogenesis as well as proliferation, invasion, and migration processes. Overall findings suggest that erianin modulates PD-L1 expression by preventing its interaction with HIF-1α while also exerting anti-tumor effects on angiogenesis and proliferation both *in vivo* and *in vitro*. The experimental design is relatively comprehensive and requires validation through clinical trials.

#### 3.1.9 Indoles

Indole compounds are widely present in nature, primarily found in natural floral oils such as jasmine, bitter orange flower, daffodils, and fragrant loran. They possess pharmacological properties including antipyretic and analgesic effects, hypotensive activity, vasodilation, and amine blocking inhibition.

Diindolylmethane (DIM), a phytonutrient and plant indole present in cruciferous vegetables such as broccoli, Brussels sprouts, cabbage, cauliflower, and kale, exhibits potential anti-androgenic and antineoplastic properties. Kandala et al. (2011) divided the cell test was divided into four groups: Control group, DIM group (50 μM), cisplatin group (10 μM), and DIM + cisplatin group. Western blot analysis revealed a significant 90% reduction in STAT-3 protein levels following combination therapy compared to individual treatment with cisplatin or DIM alone. Moreover, the expression of mcl-1 and survivin, which are regulated by STAT-3, was also downregulated. Additionally, there was a notable increase in caspase-3 and PARP cleavage. Furthermore, the results from the aortic ring assay exhibited complete inhibition of microvascular bud formation with combination treatment. In SKOV-3 cells, the combined administration of DIM and cisplatin resulted in a 50% decrease in VEGF secretion compared to only 20% inhibition observed with cisplatin alone. To summarize, DIM enhances the efficacy of cisplatin by blocking STAT-3 activation and inhibiting angiogenesis as well as metastasis. Unfortunately, the *in vivo* validation of the further mechanism underlying this combination therapy has not been conducted.

#### 3.1.10 Hormone

Melatonin is a biogenic amine present in animals, plants, and microorganisms. Yiqing et al. (2020) conducted an investigation to examine the impact of melatonin on invasion, migration, and angiogenesis of ovarian cancer cells both *in vitro* and *in vivo*. The effects of melatonin on the proliferation, invasion, and migration of SKOV3 cells were observed through MTT assay, scratch assay, and Transwell invasion assay respectively. HUVECs were induced to form tubes to evaluate the effect of melatonin on them. Western blot analysis was employed to assess the expression levels of VEGF, E-cadherin, MMP9, and Vimentin in SKOV3 cells. The results demonstrated that melatonin inhibited the invasion and migration abilities of ovarian cancer SKOV3 cells while up-regulating E-cadherin expression and down-regulating MMP-9, vimentin, and VEGF protein expressions. Furthermore, melatonin suppressed tubulogenesis in HUVECs. Immunohistochemistry was used to detect CD31 expression in ovarian cancer xenograft models which were randomly divided into two groups: Control group (200 μL normal saline) and MEL group (25 mg/kg) for 3 weeks. The results indicated a decrease in CD31 expression level, suggesting that melatonin could inhibit microvessel density. In conclusion, melatonin inhibits invasion, migration, and angiogenesis of ovarian cancer cells. The experimental design is relatively straightforward, lacking a positive control group, thereby compromising certain aspects of reliability.

#### 3.1.11 Macrolides

Rapamycin, also known as Sirolimus, is a macrocyclic lactone antibiotic derived from the bacterium *Streptomyces hygroscopicus*. It was originally isolated from the soil of Rapa Nui (Easter Island) in the Vai Atari region. The ovarian cancer SKOV-3 cells were divided into four groups: blank control group, cisplatin group (10 μmol/L), rapamycin group (40 nmol/L), and combination group (10 μmol/L cisplatin +40 nmol/L rapamycin). Cell viability, ATP production, and VEGF content in the supernatant were measured. The results demonstrated that the MTT value, ATP content, and VEGF content of the cisplatin group, rapamycin group, and combination group were significantly lower than those of the blank control group. Moreover, the combination therapy exhibited superior effects. These findings suggest that rapamycin enhances the inhibitory effect of cisplatin on ATP production and angiogenesis in ovarian cancer SKOV-3 cells. Furthermore, a synergistic effect is observed when both drugs are used together (Zhonghua, 2015). However, it should be noted that this experiment lacks certain credibility due to the absence of concentration gradient comparisons and *in vivo* animal experiments.

### 3.2 Medicinal plants and extracts targeting angiogenesis in gynecological cancer

(Table 1; Figure 3).

**TABLE 1:** Antiangiogenic therapy of medicinal plants and extracts for gynecological cancer.

Name of extract	Source (authorities and family)	Experiments	Gynecological cancer	Animal/cell model	Dose range	Hydrotropy agent	Model	Duration	Molecular mechanisms	Signal pathways	Ref.
Scutellaria barbata herba	*Scutellaria barbata* D.Don (The whole plant) [Lamiaceae]	Drug-free serum group; Medicated serum group;	Cervical cancer	HeLa/HUVEC cells	10%, 20%, 40%	—	*In vitro*	12, 24, 48 h	VEGF	—	Zhang et al. (2005)
		Drug-free serum group; Medicated serum group (100 μ)l;	Cervical cancer	Angiogenesis model of matrigel suppository in mice	100 μL	—	*In vivo*	7 days	↓:Number of angiogenesis, Erythrocyte counts		
Acanthus ebracteatus Vahl extract (AE)	*Acanthus ebracteatus* Vahl (Stem and leaves) [Acanthaceae]	Control group (untreated); control group (treatment); AE extract group	Cervical cancer	CaSki (HPV-16+)/HeL (HPV-18+)HDFs cells	10^–3_^10^4^ μg/mL	Distilled water	*In vitro*	24, 48, 72 h	↓:VEGF	—	Mahasiripanth et al. (2012)
		Control group (distilled water) p.o.; HPV-16 group (distilled water) p.o.; HPV-16 group (AE 300 mg/kg) p.o.; HPV-16 group (AE 3000 mg/kg) p.o.	Cervical cancer	CaSki (HPV-16) cells injected nude mice	300, 3,000 mg/kg	—	*In vivo*;	14, 28 days			
Senduduk fruit extract	*Melastoma malabathricum* L. (Fruit) [Melastomataceae]	Control group; Senduduk fruit extract group	Cervical cancer	HeLa cells	956 μg/mL, 1,912 μg/mL.	—	*In vitro*	24 h	↓:VEGF, TNF-α	—	Edianto et al. (2020)
Kalonji extracts	*Nigella sativa* L. (Leaves) [Ranunculaceae]	Control group; Kalonji petroleum ether extract group; ethanol extract group; water extract group	Cervical cancer	HeLa cells	100, 200, 500, 1,000, 2,000 μg/mL	DMSO	*In vitro*	24 h	↑: p53, Bax caspasase-3, ↓: VEGF, Ki67, PCNA, TOP2A	—	Maqbool et al. (2019)
Datura inoxia extract	*Datura innoxia* Mill. (Seeds) [Solanaceae]	Control group; Datura inoxia extract group	Cervical cancer	HeLa Cells	—	—	*In vitro*	—	↑: caspase-3/-9 ↓: VEGF, TNF-α	—	Pandey and Sarawati (2011)
Pllans-II	*Porthidium lansbergii* lansbergii snake venom	Control group; Pllans-II group	Cervical cancer	HeLa/HUVEC Cells	50, 100 μg/mL	—	*In vitro*	24, 48 h	↑:Bax, BCL2L1, CASP8 ↓:VEGF, Bcl-2, BAD, BIRC5	VEGF	Jimenez-Charris et al. (2019)
Penicillium sclerotiorum extract (PSE)	*Cassia fistula* L. (Leaves, stems and bark) [Fabaceae]	Control group; PSE group	Cervical cancer	HeLa cells\	5, 10 μM/mL	DMSO	*In vitro*	24 h	↑:Bax, p53, Apaf-1 ↓:Bcl-2	mitochondrial dependent pathway	Kuriakose et al. (2018)
		Control group; PSE group	Cervical cancer	CAM	10 μL/10 mM	DMSO	*In vivo*	48 h	↓:Blood vessel counts		
Transferrin-Functionalized Microemulsions Coloaded with Coix Seed Oil and Tripterine (Tf-CT-MEs)	*Coix * *lacryma-* *jobi* * var*. ma-yuen (Rom.Caill.) Stapf (Fruit) [Poaceae]; *Tripterygium wilfordii* Hook.f. (Root) [Celastraceae]	Saline group i.p. ; tripterine group (1.8 mg/kg) i.p.; CT-MEs group (44.4 mg/kg) i.p.; Tf-CT-MEs group i.p.	Cervical cancer	HeLa injected nude mice	1.8 mg/kg + 44.4 mg/kg	DMSO	*In vivo*	21 days	↑:Bax, caspase-3, ↓:CD31, Bcl-2, TGF-β1, IL-6, ki67, CCL2, TNF-α,	—	Guo et al. (2019)
Ethanol Extract from Amomum tsaoko (At-EE)	*Lanxangia * *tsao-* *ko* (Crevost & Lemarié) M.F.Newman & Škorničk. (Fruit) [Zingiberaceae ]	HUVEC group; HUVEC cells co-cultured with SKOV3 cells group; At-EE co-cultured group	Ovarian cancer	SKOV3/HUVEC/HaCaT cells	5, 10 μg/mL	DMSO	*In vitro*	24, 48 h	↑: GRP7, CHOP ↓: VEGF, IL-6, p-STAT3, NF-κB	STAT3/NF-κB	Chen et al. (2020)
		NS group; At-EE group (30 mg/kg) p.o.	Ovarian cancer	SKOV3 cells injected nude mice	30 mg/kg	Saline	*In vivo*	5 weeks	↓: CD31		
Cinnamon Extract (CE)	*Cinnamomum tamala* (Buch.-Ham.) T.Nees & C.H.Eberm. (Bark) [Lauraceae]	Vehicle control group; CE group; Cinnamaldehyde (10 mg/mL);	Ovarian cancer	HUVEC cells	32 mg/mL	Water	*In vitro*	5 h	↓:HIF-1α, VEGF,	STAT3/AKT	Zhang et al. (2017)
		Control group (water)p.o.; CE group (0.3 mg/g) p.o.	Ovarian cancer	SKOV3 cells injected nude mice	0.3 mg/g	—	*In vivo*	4 weeks	↓:CD31		
Brittle star methanol extract	Brittle star (Ophiocoma erinaceus)	Control group; Brittle star methanol extract group	Ovarian cancer	A2780cp cells	25, 50, 100 μg/mL	—	*In vitro*	24, 48 h	↓: VEGF, b-FGF	—	Baharara et al. (2015)
		Control group; Brittle star methanol extract group	Ovarian cancer	CAM	25, 50, 100 μg/mL	—	*In vivo*	12 days	↓: Blood vessel snumber and length		
Linum usitatissimum seed essential oil nanoemulsions (LSEO-NEs)	*Linum usitatissimum* L. (Seed) [Linaceae]	Control group; LSEO-NEs group;	Ovarian cancer	A2780/HFF cells/	8, 16, 32 μg/mL	—	*In vitro*	48 h	↑:Caspase-3, 8, and 9	—	(Keykhasalar et al., 2021)
		Control group; Lab control group; LSEO-NEs group;	Ovarian cancer	CAM	12.5, 25 50, 100 μg/mL	—	*In vivo*	72 h	↓: Blood vessel snumber and length		
Polypeptide extract from scorpion venom (PESV)	East Asian scorpion scorpion venom	Control group; PESV group	Ovarian cancer	SKOV3 cells	400 mg	Saline	*In vitro*	—	↓:HPA, VEGF	—	Xiaohui et al. (2012)
		Control group; PESV group	Ovarian cancer	SKOV3 cells	12.5, 25, 50, 100, 200 μg/mL	PBS	*In vitro*	48 h	↓:VEGF ↑:TSP-1	—	Yan et al. (2012)
Zerumbone pendant derivative (ZPD)	*Zingiber zerumbet* (L.) Roscoe ex Sm. (Rhizome) [Zingiberaceae]	Control group; Paclitaxel groups (10 nM); ZPD group	Cervical cancer	HeLa cells	2.5, 5, 10 μM	—	*In vitro*	24 h	↑: Bax ↓:VEGF, MMP-2, MMP-9, Bcl-2	—	Saranya et al. (2017)
Asparagus officinalis extract (ASP)	*Asparagus officinalis* L. (stem) [Asparagaceae]	Control group; ASP group	Ovarian cancer	OVCAR5/SKOV3 cells	0.1, 1, 5 mg/mL	—	*In vitro*	14, 18, 24, 48 h	↑:cleaved-caspase 3, -8, - 9, PERK, ATF4, PDI, BiP, Calnexin, ROS ↓:VEGF, p-AKT, p-S6, 7BCL-XL, MCL-1, CDK4, CDK6	AKT/mTOR	Xu et al. (2021)
		Control group (HFD); HFD + ASP group; LFD group; LFD + ASP group	Ovarian cancer	Injected Ad5-CMV-Cre KpB mouse	200, 800 mg/kg	—	*In vivo*	4 weeks	↑:p-AMPK, ↓:VEGF, p-S6, Ki-67		
Cell Wall-Short Chain Carbohydrate (CW-SCC)	Plant primary cell wall	CaSki + vehicle group p.o.; CaSki group p.o.	Ovarian cancer	CaSki cells injected nude mice	60, 120 mg/kg	—	*In vivo*	30 days	↓:VEGF, COX-2/EGFR, CD31	—	(2017)
Okra seed Extract	*Abelmoschus esculentus* (L.) Moench (Seed) [Malvaceae]	Control group; Okra seed Extract groups; isoquercitrin (28.9 μg/mL)	Cervical cancer	HeLa cells	100, 250, 500 μg/mL	DMSO	*In vitro*	24, 48 h	↓:VEGF	—	Chaemsawang et al. (2019)
A water-soluble polysaccharide (PTP)	*Polygala tenuifolia Willd*. (Roots) [Polygalaceae]	Negative control group (PBS) p.o.; PTP group p.o.	Ovarian cancer	SKOV3 cells injected nude mice	10, 20, 40 mg/kg	D_2_O	*In vivo*	2 weeks	↓:EGFR, VEGF, CD34	—	Yao et al. (2018)
TWY extracts	*Salvia miltiorrhiza* Bunge (Root) [Lamiaceae]; *Tripterygium wilfordii* Hook.f. (Root) [Celastraceae]; *Sparganium stoloniferum* (Buch.-Ham. ex Graebn.) Buch.-Ham. ex Juz. (Root) [Typhaceae]; *Curcuma aromatica* Salisb. (Root) [Zingiberaceae]; *Panax notoginseng* (Burkill) F.H.Chen (Root) [Araliaceae]; *Carthamus tinctorius* (Flowers) [Asteraceae] and other 16 kinds of Chinese herbal medicine extracts	TWY high, medium and low dose groups (7 mg/mL)i.g.; TWY Prevention group (4 g/kg·d) i.g.; TWY Prevention group (4 g/kg·d) i.p.; CTX group (0.02 g/kg·d) i.p.; KLT group (0.25 g/kg·d) i.p.; FYT group (0.04 g/kg·d) i.g.	Cervical cancer	U14 cells injected KM species of mice	2, 4, 8 g/kg·d	saline	*In vivo*	14 days, 21 days	↑: TSP-1 ↓:VEGF, KDR, MVD	—	Yong (2006)
		Control group; TWY group	Cervical cancer	CAM	0.4375, 0.875, l.75, 3, 7 mg/mL	—	*In vivo*	12 days	↓: Blood vessel snumber and length		

The majority of natural product extracts are derived from plants, algae, fungi, lichens or animals and encompass a diverse array of bioactive compounds that typically exhibit multi-target activity. The extraction of active constituents is a crucial stage in the development of these natural product. Traditional extraction methods encompass maceration, percolation, decoction, reflux, among others. However, their drawback lies in the lengthy extraction time and low yield. Modern extraction techniques typically enhance the target product’s extraction rate through pressure and other auxiliary means such as supercritical fluid extraction, pressure liquid extraction, and microwave-assisted extraction. These methods offer advantages including solvent conservation and shortened processing time. Given the intricate composition of natural product extracts, many exhibit unfavorable physical properties like poor flowability and high moisture absorption tendency-particularly those containing polysaccharides, tannins, small molecular alkaloids or similar components. Numerous studies have demonstrated that many natural product extracts can synergistically interact with Western medicines (Ling et al., 2022; Guoqiang et al., 2023).

For example, *Acanthus ebracteatus* Vahl (AE), commonly known as Sea Holly, is a versatile medicinal plant found in various southeast Asian countries such as Thailand, the Philippines, Indonesia, and others (Somchaichana et al., 2012; Prasansuklab and Tencomnao, 2018). The effect of AE extract on the proliferation and angiogenesis of HPV-16 DNA cervical cancer cells, as well as on xenograft tumors in nude mice, has been observed in studies. *In vitro* experiments were conducted to evaluate the impact of AE on diverse cell lines, such as CaSki (HPV-16 positive), HeLa (HPV-18 positive), and human dermal fibroblasts (HDFs). The results revealed that AE displayed dose-dependent inhibitory effects across multiple cell types over time. The CaSki cells (HPV-16 positive) were injected into the dorsal region of nude mice to establish an *in vivo* model. Following successful modeling, AE extract was administered orally at doses of 300 mg/kg or 3,000 mg/kg for a duration of either 14 or 28 days. The distribution of tumor microvessels and capillaries was visualized using laser confocal microscopy, while VEGF expression was assessed through immunohistochemistry. The findings revealed a linear correlation between the reduction in tumor volume and the decrease in tumor capillary distribution. Treatment with a high dose of AE extract significantly suppressed VEGF expression. In conclusion, this study provides novel evidence that AE crude extract possesses inhibitory effects on cervical cancer growth and angiogenesis in a mouse model transplanted with CaSki cells (Mahasiripanth et al., 2012). However, it is worth noting that this experiment lacked a positive control group, had a simplistic design for the *in vitro* experiments, limited assessment of relevant indicators, and did not identify specific components within the AE extract responsible for its therapeutic effects.

Amomum tsaoko, a member of the ginger family, exhibits anti-insect infestation properties as well as anti-liver cancer cell (Yang et al., 2010; Wang et al., 2014; Zhang et al., 2016) and anti-tumor effects. Ginger plant has demonstrated promising anticancer effects on various types of tumors (Al-Abbasi et al., 2016; Lee et al., 2016; Malami et al., 2017; van Die et al., 2017). The At-EE is a hydroalcoholic extract derived from *Lanxangia tsao-ko.* How does At-EE inhibit angiogenesis? Chen et al. (2020) observed that At-EE did not induce apoptosis in HUVEC cells and HaCaT cells, nor did it affect the migration and angiogenic abilities of HUVEC cells. It is suggested that At-EE has no direct impact on vascular endothelial cells and normal cells. Subsequently, co-culture experiments were conducted to investigate the potential influence of At-EE on the crosstalk between endothelial cells and tumor cells. The findings revealed an enhanced invasion ability of HUVEC after co-culturing with SKOV3 cells, indicating that At-EE may modulate the movement and formation of vascular endothelial cells through regulation of cytokine secretion by tumor cells. Furthermore, RT-qPCR, Elisa, and Western blot analyses demonstrated that At-EE reduced the expression levels of VEGF, IL-8, and IL-6; suppressed NF-kB and p-STAT3 activation; while increasing GRP78 and CHOP expression levels. These results suggest that At-EE exerts its inhibitory effects on p-STAT3/NF-kB/IL6 signaling loop as well as VEGF pathway by inducing endoplasmic reticulum stress. The *in vivo* experiment involved the random division of nude mice into two groups (8 mice/group), with one group receiving treatment with At-EE (30 mg/kg) and the other group receiving normal saline for a duration of 4 weeks. Immunohistochemistry was used to detect CD31 expression, which was found to be significantly lower in the At-EE treatment group compared to the control group. Overall, this study provides novel evidence that At-EE can inhibit ovarian cancer cell proliferation by suppressing angiogenesis both *in vitro* and *in vivo*. Furthermore, it demonstrates that p-STAT3/NF-kB/IL-6 and VEGF form a cascade amplification loop promoting angiogenesis in ovarian cancer, which can be disrupted through induced ER stress. Chen et al. were the first to demonstrate that At-EE induces endoplasmic reticulum stress, leading to the inhibition of p-STAT3/NF-kB/IL-6 and VEGF signaling pathways, thereby suppressing angiogenesis and tumor growth. Importantly, high concentrations of amomum did not affect normal cells. The experimental design was comprehensive, and a co-culture experiment was incorporated into the *in vitro* study. The only limitation observed was the absence of a positive control group, necessitating further investigations to ensure data reliability.

Brittle stars, which belong to the ophiuroidea family, constitute the largest group of echinoderms and have gained attention due to their remarkable capacity for arm regeneration. The primary bioactive metabolites identified in this family include chlorinated biphenyls, triterpenes, and polycyclic hydrocarbons. The methanol extracts of brittle stars have been demonstrated to possess inhibitory effects on ovarian cancer progression and exhibit anti-angiogenic potential in studies (Baharara et al., 2015). The MTT assay was utilized to evaluate the inhibitory effects of brittle star extracts on A2780cp cell proliferation *in vitro*, while RT-qPCR was employed to assess transcriptional expression of VEGF and b-FGF. *In vivo* experiments involved incubating 40 fertilized ross eggs with varying concentrations (25, 50, and 100 μg/mL) of brittle star extract followed by imaging using a light-stereomicroscope. ImageJ software facilitated measurement of blood vessel number and length. Results indicated that methanol extracts from brittle stars inhibited A2780cp cell proliferation in a dose- and time-dependent manner, as well as reduced VEGF and beta-FGF expression levels. These findings demonstrate the antiangiogenic properties of methanol extracts from brittle snake skin both *in vitro* and *in vivo*. The present experiment demonstrates the antiangiogenic properties of natural marine products and offers novel insights into the marine ecosystem as a potential source of metabolites with anticancer activity. However, it is worth noting that the experimental design employed in this study is relatively simplistic, lacking a positive control group.

The *Linum usitatissimum* L. seed essential oil (LSEO) contains a diverse range of phytochemicals, including phenols, flavonoids, and lignin. Keykhasalar et al. (2021) initially developed a nanoemulsion encapsulation technique to create a safe and natural drug delivery system for LSEO, enabling the investigation of its apoptotic and antiangiogenic activities. The LSEO nanoemulsion (LSEO-NE_S_) was prepared using ultrasonic technology, and its particle size, droplet morphology, and stability were characterized. The cytotoxicity of LSEO-NEs was investigated by assessing the viability of human ovarian cancer A2780 cells and HFFS cells. Apoptotic activity was evaluated by measuring the gene expression levels of caspase-3, caspase-8, and caspase-9. Furthermore, the anti-angiogenic potential was assessed using a CAM assay. The results demonstrated that LSEO-NEs exhibited significant dose-dependent cytotoxic effects on A2780 cells while not affecting HFFS cells. Additionally, the reduction in vessel length and number observed in the CAM assay indicated that LSEO-NE possessed antiangiogenic activity. In conclusion, this experiment confirmed the tumor cell-selective apoptotic effects and antiangiogenic properties of LSEO-NEs. However, further analysis with other ovarian cancer cell lines is necessary to validate their potential anticancer effects on human ovarian cancer cells.

Currently, there is a wide range of natural product extracts available that have demonstrated significant efficacy in inhibiting tumor angiogenesis both *in vivo* and *in vitro* experimental studies (Table 1), thereby effectively impeding the progression of gynecological cancer.

## 4 Discussion and perspective

**FIGURE 4:**
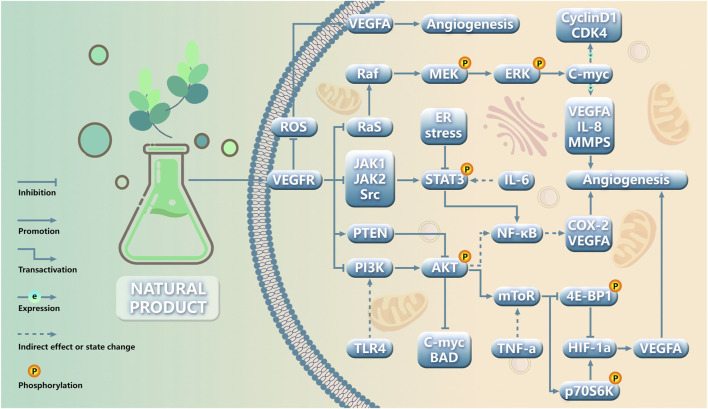
Schematic role of natural products in gynecological cancer angiogenesis.

In this review, we conducted a comprehensive search and screening of five databases, ultimately identifying 63 natural products that have demonstrated inhibitory effects on angiogenesis in gynecological cancer. Regrettably, there is currently no available literature specifically addressing vulvar cancer, uterine sarcoma, and choriocarcinoma. The VEGF pathway has emerged as the primary target for impeding tumor neovascularization. The pathways of natural products targeting angiogenesis in gynecological cancer were classified through comparative analyses (Figure 4): (1) VEGF-targeted approaches, focusing on this pivotal regulator of angiogenesis; (2) PI3K/AKT-targeted strategies, which play a crucial role in tumor angiogenesis regulation; (3) HIF-1α-mediated regulation of tumor angiogenesis; (4) ERK-targeted interventions for regulating tumor angiogenesis; (5) Others.

Most of the research data in this review are derived from *in vitro* and *in vivo* experiments, with limited data from clinical trials. Although natural products have demonstrated significant potential in the treatment of gynecologic cancers, concerns regarding certain side effects persist. To maintain optimal efficacy of specific biological activities, an increase in dosing frequency is required, resulting in reduced patient adherence and potentially cumulative drug toxicity. Therefore, further clinical trials and efficacy evaluations are necessary to mitigate adverse effects associated with the utilization of these reviewed natural products as a therapeutic approach.

It is worth considering that some natural products exhibit limited oral bioavailability. For instance, the medicinal applications of certain oily components derived from plants, such as *curcuma zedoaria* volatile oil and *coix* seed oil, are constrained due to their low biocompatibility and solubility in the bloodstream. However, drug delivery systems have been developed as effective optimizers to enhance their biocompatibility and accessibility through nanoemulsification methods (Yukuyama et al., 2017). Nanoemulsions consist of droplets (up to 200 nm) dispersed in aqueous or hydrophobic solutions and encompass polar or non-polar compounds (Sadurni et al., 2005). By serving as suitable carriers, nanoemulsions augment the solubility and biocompatibility of nonpolar therapeutic agents. They find extensive application in medicine (Singh et al., 2017). Plant essential oil nanoemulsions like *Syringa vulgaris*, *Thymus mongolicus* and *Prunus cerasus* have been employed as targeted anticancer drugs with reduced side effects (Kubatka et al., 2019; Maragheh et al., 2019; Nirmala et al., 2019). Nanodrug delivery systems can enhance solubility, improve physicochemical stability and bioavailability of poorly soluble drugs, optimize delivery efficiency, and mitigate toxicity.

**FIGURE 5:**
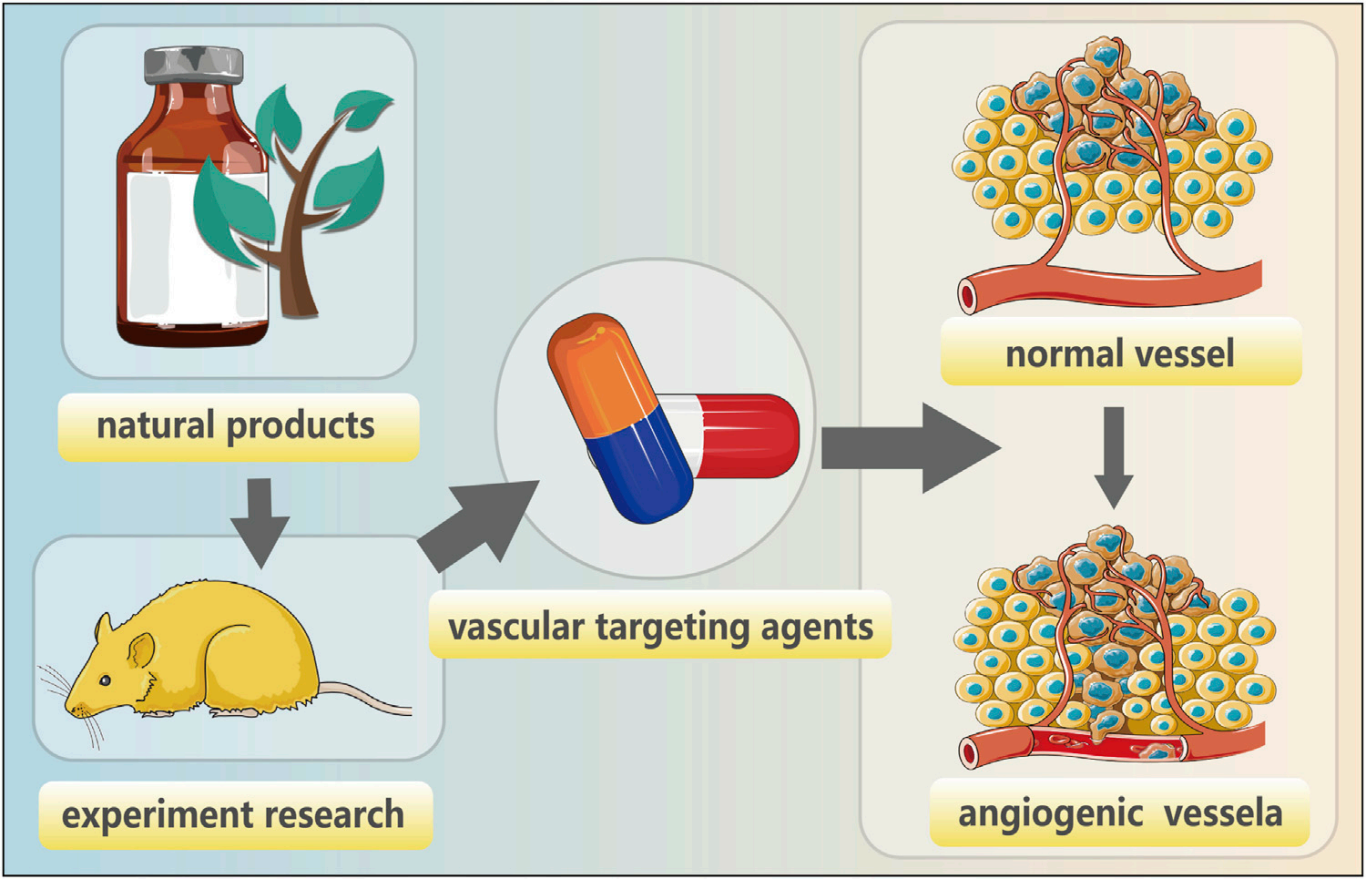
Steps of natural products in anti-angiogenesis therapy for gynecological cancer.

In summary, natural products have emerged as a significant reservoir for the development of effective antitumor drugs in comparison to chemical drugs due to their diverse structures, low toxicity, and unique mechanisms of action. These natural products encompass animals (including marine organisms), plants, and microorganisms (Figure 5) and have demonstrated potential in reducing the risk of gynecologic cancer. They comprise a wide array of bioactive compounds such as flavonoids, alkaloids, polysaccharides, glycosides, etc. The multifaceted and intricate anticancer effects exhibited by these constituents necessitate further investigations to ascertain the viability of natural products as promising targets for treating gynecologic cancers.

Finally, a comprehensive understanding of the mechanism of action and therapeutic effects of natural products in targeted anti-gynecological angiogenesis is crucial. This knowledge provides robust support for elucidating the intricate interplay between natural products and anti-tumor angiogenesis, as well as informing clinical treatment strategies and facilitating new drug development.

## Abbreviations

VEGFR, Vascular endothelial growth factor receptor; PI3K, Phosphoinositide3-kinase; Akt, Protein kinase B; ERK, Extracellular regulated protein kinases; HIF-1α, Hypoxia-inducible factor 1 α; mTOR, mammalian target of rapamycin; EGFR, Epidermal growth factor receptor; MMP-2, Matrix metalloproteinase 2; MMP-9, Matrix metalloproteinase 9; NF-κB, Nuclear factor-k-gene binding k; TNF-α, Tumor Necrosis Factor-α; Bcl-2, B-cell lymphoma-2; Bax, BCL2-associated X protein; IL-6, Interleukin 6; COX-2, Cyclooxygenase 2.
