# Supplementary material for: Natural products’ antiangiogenic roles in gynecological cancer
Source: Front Pharmacol. 2024 May 1;15:1353056. doi: 10.3389/fphar.2024.1353056 (PMC11094279; doi:10.3389/fphar.2024.1353056)
Supplement: Supplementary file 1 [file Table1.docx]

**TABLE S1 Natural compounds antiangiogenic treatment for gynecological cancer**

| **Classifications** | **Name** | **Source**  **(authorities and family )** | **Experiments** | **Gynecological cancer** | **Animal/cell model** | **Dose range** | **Hydrotropy agent** | **Model** | **Duration** | **Molecular mechanisms** | **Signal pathways** | **Ref.** |
| --- | --- | --- | --- | --- | --- | --- | --- | --- | --- | --- | --- | --- |
| **Terpenoid** | Triptolide (TPL) | *Tripterygium wilfordii* Hook.f. (Root) [Celastraceae] | Contol group;  (saline 0.2 ml)  i.p.; DDP group (40 ug) i.p.; Triptolide high, middle and low dose group i.p. | Endometrial cancer | HEC-1B cells injected nude mice | 2, 4, 8 μg | saline | *In vivo* | 15 days | ↓: Bcl-2, VEGF | / | (Jian, 2012) |
|  |  |  | Control group  (Saline 50 ml/kg) i.p.; TPL group i.p.; DDP group  （4 mg/kg）i.p.;  TPL+DDP i.p. | Ovarian cancer | SKOV3 and DDP cells injected nude mice | 0.15mg/kg | 0.9% NaCl | *In vivo* | 14 days | ↓: VEGF, CD31,  CD105, CD16, CD56, TNF-a  ↑:IL-2, CD4+,  CD8+ | / | (Xiaobing, 2016) |
|  | Artemisinin | *Artemisia caruifolia* Buch.-Ham. ex Roxb. (Stem and leaves)  [Asteraceae] | Control group; Artemisinin group | Cervical cancer | ME-180/HeLa cells | 75, 150,  300 μM | / | *In vitro* | 24, 48, 72, 96 h | ↑: P53,  ↓:ERα, VEGF, hTR, E6, E7， Cyclin-D1, CDK4, P21, | / | (Mondal et al., 2015) |
|  | Artesunate  (ART) | *Artemisia caruifolia* Buch.-Ham. ex Roxb. (Stem and leaves)  [Asteraceae] | Control group（saline）i.m.; Tumor control group（saline）i.m.; ART group i.m.; DDP group  （2 mg/kg）i.p. | Cervical cancer | U14 Cells injected nude mice | 2 mg/kg | 5%NaHCO_2_ | *In vivo* | 7 days | ↓: COX-2,  VEGF, MVD | / | (Zhaoxi, 2017) |
|  |  |  | Negative control group; Positive control group; ART group; | Ovarian cancer | CAOV3Aortic rings were cultured | 3, 6,  12 μmol /L | / | *In vitro* | 15 days | ↓: Microvessels number, VEGF | / | (Sumei et al., 2011) |
|  |  |  | NS group; CAOV3 cells group ( 5×10^6^ cells/100 μl); ART group | Ovarian cancer | CAM | 3, 6,  12 μmol /L | / | *In vivo* | 12 days | ↓: Microvessels number |  |  |

**TABLE 1-1 *(Continued)* Natural compounds antiangiogenic treatment for gynecological cancer**

| **Classifications** | **Name** | **Source**  **(authorities and family )** | **Experiments** | **Gynecological cancer** | **Animal/cell model** | **Dose range** | **Hydrotropy agent** | **Model** | **Duration** | **Molecular mechanisms** | **Signal pathways** | **Ref.** |
| --- | --- | --- | --- | --- | --- | --- | --- | --- | --- | --- | --- | --- |
| **Terpenoid** | Artesunate  (ART) | *Artemisia caruifolia* Buch.-Ham. ex Roxb. (Stem and leaves)  [Asteraceae] | NS group; ART group | HUVEC/Hela/HO-8910 cells | Ovarian cancer | 2.5, 12.5,  50 μmol /L | DMEM | *In vitro* | 48, 72 h | ↓:VEGF, CD31, KDR/flk-1, | / | (Chen et al., 2004) |
|  |  |  | NS group, ART High, middle and low dose group i.h. | HO-8910 cells injected BALB/C nude mice | Ovarian cancer | 10, 50,  100 mg/kg | Saline | *In vivo* | 15 days | ↓:VEGF, CD31, KDR/flk-1, |  |  |
|  | Betulin | *Betula pendula subsp. mandshuric*a (Regel) Ashburner & McAll. (Bark) [Betulaceae] | Control group; Betulin group; | HeLa cells | Cervical cancer | 3, 10, 30 μM | Distilled water | *In vitro* | 24 h | / | **/** | (Dehelean et al., 2012) |
|  |  |  | Control group; DMSO group (0.5%); Betulin DMSO group; | CAM | Cervical cancer | 30 µM | Distilled water | *In vivo* | 10 days | ↓:Blood vessel counts |  |  |
|  | Ginsenoside Rg3 | *Panax ginseng* C.A.Mey. (Roots and rhizomes) [Araliaceae] | Control group ( saline 0.5 ml/d) p.o.; DDP group (5 mg/kg) i.p.; Rg3 (5 mg/kg) p.o.; Rg3 (5 mg/kg p.o) +DDP group (5 mg/kg i.p.) | Cervical cancer | Hela cells injected nude  mice | 5 mg/kg | saline | *In vivo* | 5 weeks | ↓:CD31, PCNA | **/** | (Yingchun, 2007) |
|  |  |  | Normal group; Control group (PBS 0∙4 ml) p.o.; Rg3 group (PBS 0∙4 ml )+ Rg3 (0∙75 mg/ml) p.o. | Ovarian cancer | SKOV3 cells injected CB-17scid mice | 0∙75 mg/ml | PBS | *In vivo* | 26 days | ↓: VEGF, CD34 | / | (Zimin et al., 2002) |
|  | Paclitaxel (PTX) | *Taxus wallichiana* Zucc. (Roots, leaves and bark) [Taxaceae] | NC group; alphastatin group (0.25 mg /kg)i.p.; PTX group (20 mg /kg) i.v.; Combined treatment group | Ovarian cancer | SKOV3 cells injected nude mice | 20 mg /kg | PBS | *In vivo* | 12 days | ↓: VEGF, AKT, PI3K, MVD | VEGF/AKT/  PI3K | (Chunyan et al., 2018) |

**TABLE 1-2 *(Continued)* Natural compounds antiangiogenic treatment for gynecological cancer**

| **Classifications** | **Name** | **Source**  **(authorities and family )** | **Experiments** | **Gynecological cancer** | **Animal/cell model** | **Dose range** | **Hydrotropy agent** | **Model** | **Duration** | **Molecular mechanisms** | **Signal pathways** | **Ref.** |
| --- | --- | --- | --- | --- | --- | --- | --- | --- | --- | --- | --- | --- |
| **Terpenoid** | Atractylenolide 1 | *Atractylodes macrocephala* Koidz. (Rootstock) [Asteraceae] | PTX (0.001-0.05 μmo/L); LPS (0.01-10ug/m1);  Atractylenolide1 (1-100 μmo/L) | Ovarian cancer | SKOV-3/A2780 cells | 10-100 μmo/L | DMSO | *In vitro* | 48 h | ↓:VEGF | TLR4/  MyD88+ | (Wen, 2011) |
|  | Glycyrrhetinic acid (GA) | *Glycyrrhiza uralensis* Fisch. ex DC. (Root and stem)  [Fabaceae] | Control group;  GA group | Ovarian cancer | A2780/HUVEC cells | 5, 10, 20 μM | / | *In vitro* | 24,48 h | ↓:VEGFR2, mTOR, p-mTOR, AKT, p-Akt, p38,  ERK1/2, p-ERK1/2, MEK1/2, JNK1/2 | / | (Li et al., 2020) |
|  |  |  | Control group;  GA group | Ovarian cancer | Rat aortic ring angiogenesis model | 10, 20, 40 μM | / | *In vitro* | 7 days | ↓:VEGF |  |  |
|  |  |  | Control group (0.5% CMC-Na insaline buffer);  GA group p.o.; | Ovarian cancer | A2780 cells injected nude mice; | 10, 30 mg/kg | 0.5% CMC-Na | *In vivo* | 10 days | ↓:CD31 |  |  |
|  |  |  | Control group;  GA group | Ovarian cancer | Zebrafish embryos | 30, 100,  300 μM | / | *In vivo* | 12 h | Number intact ISVs |  |  |
|  | Gossypol | *Gossypium barbadense* L. (Flower)  [Malvaceae] | Control group;  Cisplatin group; (5 μM );  Gossypol group;  Gossypol + Cisplatin group (10μM + 5μM); | Ovarian cancer | OVCAR-3 cells | 10 μM | / | *In vitro* | / | ↑:caspase-3/-7  ↓:Ang-1 | / | (Isbir, 2009) |
|  | Ascochlorin | *Ascochyta viciae* Libert (the fungus A. viciae) | Control group; EGF (20 ng/ml) group;  Ascochlorin group; | Cervical cancer | CaSki cells | 200 μM | / | *In vitro* | 12, 24 h | ↓:HIF-1α, VEGF, P-EGFR, PERK, P-p70S6K | EGFR/ERK/  p70S6K | (Jeong et al., 2012) |
|  |  |  | Control group; EGF (500 ng/ml) group; Ascochlorin group; | Cervical cancer | CaSki cells injected  C57BL/6N mice | 10, 30 mM | / | *In vivo* | 7 days | ↓:HIF-1α, VEGF, |  |  |

**TABLE 1-3 *(Continued)* Natural compounds antiangiogenic treatment for gynecological cancer**

| **Classifications** | **Name** | **Source**  **(authorities and family )** | **Experiments** | **Gynecological cancer** | **Animal/cell model** | **Dose range** | **Hydrotropy agent** | **Model** | **Duration** | **Molecular mechanisms** | **Signal pathways** | **Ref.** |
| --- | --- | --- | --- | --- | --- | --- | --- | --- | --- | --- | --- | --- |
| **Quinones** | Emodin | *Rheum palmatum* L. (Roots)  [Polygonaceae] | Control group (DMSO); Emodin low- dose group (20 mg/kg) p.o.; Emodin high- dose group (40 mg/kg) p.o.;  Cisplatin group  (3 mg/kg) i.p | Cervical cancer | U14 cells injected nude mice | 20 mg/kg,  40 mg/kg, | DMSO | *In vivo* | 26 days | ↑: Bax  ↓:HIF-1α, VEGF, CD34,, MIF, Bcl-2 | HIF-1α/  VEGF | (Zhang et al., 2015) |
|  | Plant-*Nigella sativa* (NS) and Thymoquinone (THM) | *Nigella sativa* L. (Seed)  [Ranunculaceae] | Control group; DTX (2 μM)  PTX (2 μM)  NS group;  THM group;  DTX+NS/THM group; PTX+NS/THM group; | Ovarian cancer | SKOV-3 cells | NS 50 μg,  THM 50 μM | / | *In vitro* | 24 h | ↑:IL-8, Bax, ↓:TNF-α, VEGF, Bcl-2, | IL-8/TNF-α/  VEGF/Bax/Bcl-2 | (Mathur et al., 2016) |
|  | Dihydrotanshinone I（DHT） | *Salvia miltiorrhiza* Bunge (Root)  [Lamiaceae] | Control group;  DHT group;  TNF group | Cervical cancer | HeLa cells, | 1, 3,10 μM | DMSO | *In vitro* | 12, 24 h | ↓:IκBα, p65, cIAP-1, FLIP, COX-2, IL-6,  MMP-9, VEGF, TNF-α,  MCP1 | NF-κB | (Wang et al., 2015) |
|  |  |  | Control group; DHT group p.o.; Vehicle group | Cervical cancer | HeLa cells injected nude mice | 15 mg/kg | Saline | *In vivo* | 35 days | ↓:TNF-α |  |  |
| **Glucosides** | Lentinan(LNT) | *Phaseolus vulgaris* L.(Fruit) [Fabaceae] | Control group; LNT group | Cervical cancer | HeLa cells | 10,25,50,  100,200,  400 μg/mL | / | *In vitro* | 24, 48, 72 h | ↑: p53, Bax,  caspase-3, ROS  ↓: Bcl-2, MDM2  PARP1, STAT3, VEGF | PI3K/Akt/  mT0R | (Hui, 2016) |
|  |  |  | Control group; (0.2 ml saline) i.p.; LNT group  (1 mg/kg) i.p. | Cervical cancer | HeLa Cells  injected nude  mice | 1 mg/kg | saline | *In vivo* | 20 days |  |  |  |

**TABLE 1-4 *(Continued)* Natural compounds antiangiogenic treatment for gynecological cancer**

| **Classifications** | **Name** | **Source**  **(authorities and family )** | **Experiments** | **Gynecological cancer** | **Animal/cell model** | **Dose range** | **Hydrotropy agent** | **Model** | **Duration** | **Molecular mechanisms** | **Signal pathways** | **Ref.** |
| --- | --- | --- | --- | --- | --- | --- | --- | --- | --- | --- | --- | --- |
| **Glucosides** | Theasaponin E_1_ (TSE_1_) | *Camellia sinensis* (L.) Kuntze (Seeds)  [Theaceae] | Control group;  TSE_1_ group | Ovarian cancer | OVCAR-3/A2780/CP70/IOSE-364 cells | 1, 2.5, 5 µg/mL | / | *In vitro* | 24 h | ↓: HIF-1α, VEGF | / | (Jia et al., 2017) |
|  |  |  | Control group;  TSE_1_ group (2 µM);  wortmannin group (100 nM); DAPT group (80 µM); TSE_1_+  wortmannin  T/DAPT (80 µM) | Ovarian cancer | OVCAR-3/A2780/CP70cells | 1, 2, 4 µM | / | *In vitro* | 24 h | ↑：Bax,Bcl-xL, pro/cleaved-Caspase-9  ↓: VEGF, HIF-1α,PTEN, p-ATM, p-Akt Akt, p-mTOR, mTOR, p-p70S6K,  p70S6K, Dll4, NICD,4E-BP1p-4E-BP1,  Jagged1 | Notch1/ATM/  PTEN/Akt/ mTOR /HIF-1α | (Li et al., 2021) |
|  |  |  | Control group;  TSE_1_ group | Ovarian cancer | CAM | 4 µM | / | *In vivo* | 15 days | ↓: VEGF |  |  |
|  | Securidaca saponin | *Securidaca longepedunculata* Fresen. (Root) [Polygalaceae] | Control group;  Securidaca saponin 4A3 group; Securidaca saponin 4A4 group; | Cervical cancer | CaSki cells | 7.03 μg/mL, 16.39 μg/mL | / | *In vitro* | 48, 72 h | ↓:VEGFA, MCL-1, BCL2L1, AKT-3、MALAT1 | PI3k-AKT/ mTOR/ NF-kB | (Obasi et al., 2018) |
| **Alkaloids** | Sanguinarine | *Macleaya cordata* (Willd.) R. Br.  (Whole grass with roots)  [Papaveraceae] | Control group；Sanguinarine group； | Cervical cancer | HeLa/Siha cells | 0.5, 0.75, 1, 2, 3 µmol/L | DMSO | *In vitro* | 8, 16, 24,  48 h | ↓:α-catenin, β-catenin, γ-catenin，MMP-2，MMP-9, VEGF | Cytochrome C→Caspase-9→Caspase-3→PARP / MAPK | (Jiaying, 2013) |
|  |  |  | Control group (0.1ml saline) i.p.; Cisplatin group (5 mg/kg）i.p.;Sanguinarine low-dose group  i.p.;Sanguinarine high dose group  i.p. | Cervical cancer | HeLa cells  injected nude  mice | 1.25 mg/kg  2.5 mg/kg | saline | *In vivo* | 28 days | ↑: Bax ↓:VEGF, EGFR, Bcl-2,  CD34, Ki-67, |  |  |

**TABLE 1-5 *(Continued)* Natural compounds antiangiogenic treatment for gynecological cancer**

| **Classifications** | **Name** | **Source**  **(authorities and family )** | **Experiments** | **Gynecological cancer** | **Animal/cell model** | **Dose range** | **Hydrotropy agent** | **Model** | **Duration** | **Molecular mechanisms** | **Signal pathways** | **Ref.** |
| --- | --- | --- | --- | --- | --- | --- | --- | --- | --- | --- | --- | --- |
| **Alkaloids** | Crinamine | *Crinum asiaticum* L. ( Bulbs )  [Amaryllidaceae] | DMSO group; Cisplatin group  (20 µM);  Crinamine group | Cervical cancer | SiHa cells | 8, 16 µM | DMSO | *In vitro* | 4, 24 h | ↓:VEGFA, AKT1, BCL2L1, CCND1, CDK4, PLK1, RHOA | / | (Khumkhrong et al., 2019) |
|  |  |  | DMSO group; Crinamine group |  | Zebrafish  Embryos | 4, 8, 16 µM | / | *In vivo* | 2 days | ↓:VEGFA |  |  |
|  | Berberine (BBR) | Coptis chinensis Franch. (Rhizome)  [Ranunculaceae] | Control group; BBR group | Ovarian cancer | SKOV3 cells | 5, 10, 25, 50, 100 mmol/ml | / | *In vitro* | 48 h | ↓:VEGF, IL-8,  S1P, S1PR1,  MMP-9, MMP-2 | S1P /S1PR1 | (Furong et al., 2023) |
|  | Chaetoglobosin K | *Zea mays* L.  (Ear and stalk) [Poaceae] | Control group;  CHK group | Ovarian cancer | A2780/CP70, OVCAR-3 /HUVEC cells | 0.5, 1,  2 μmol/L | DMSO | *In vitro* | 24 h | ↓:AKT，p-AKT, HIF-1α, VEGF,  Mtor, p-mTOR, | AKT/HIF/  VEGF | (Luo et al., 2013) |
|  |  |  | Control group;  CHK group |  | CAM | 1, 2 μmol/L | DMSO | *In vivo* | 5 days | ↓:Blood vessel count |  |  |
|  | Oxostephanine | *Stephania dielsiana* Y.C.Wu (Leaves)  [Menispermacea] | Control group;  VX-680 group (0.2 µM);  Oxostephanine group; | Ovarian cancer | OVCAR-8/HUVEC/ hFBs cells | 0.04, 0.2, 1, 5, 25 µM | / | *In vitro* | 24, 48 h | ↓: VEGF-A, HGF, FGF-2, Tube lengths number and tube branching  points | / | (Tran et al., 2022) |
|  | Tetramethylpyrazine (TMP) | *Conioselinum anthriscoides*(H.Boissieu) Pimenov & Kljuykov (Root)  [Apiaceae] | Contro group;  VEGF group (20 ng/ml); PTX group (100 nM ); TMP group (100µM ); PTX+TMP group (100 nM +100 μM) | Ovarian cancer | A2780/SKOV3/ HUVEC cells | 100 µM | DMSO | *In vitro* | 24 h | ↑:caspase 3, -9,  cleavaved - parp  ↓:p-Erk1/2, p-Akt, p-p38, | ERK1/2 and Akt | (Zou et al., 2019) |
|  |  |  | Contro groupl,  PTX treated group(5 mg /kg); TMP-treated group (60 mg /kg);PTX+TMP-treated group; | Ovarian cancer | A2780 cells injected nude mice | 60 mg /kg | Polyoxyethylene castor oil and ethanol mixture | *In vivo* | 12 days | ↓:Ki-67, CD31, , VEGF |  |  |

**TABLE 1-6 *(Continued)* Natural compounds antiangiogenic treatment for gynecological cancer**

| **Classifications** | **Name** | **Source**  **(authorities and family )** | **Experiments** | **Gynecological cancer** | **Animal/cell model** | **Dose range** | **Hydrotropy agent** | **Model** | **Duration** | **Molecular mechanisms** | **Signal pathways** | **Ref.** |
| --- | --- | --- | --- | --- | --- | --- | --- | --- | --- | --- | --- | --- |
| **Polyphenols** | Tetrahydrocurcumin (THC) | *Curcuma longa* L. (Root) [Zingiberaceae] | Control +vehicle (corn oi); Control +THC (500 mg/kg);  CaSki + vehicle (corn oi); CaSki + THC (100 mg/kg); CaSki + THC (300 mg/kg); CaSki + THC (500 mg/kg) | Cervical cancer | CaSki cells injected nude mice | 100, 300,  500 mg/kg | / | *In vivo* | 30 days | ↓:VEGF, CD31, VEGFR-2, HIF-1 | HIF-1α/VEGF/VEGFR-2 | (Yoysungnoen et al., 2015) |
|  | Curcumin (CUR) | *Curcuma longa* L. (Rhizome) [Zingiberaceae] | Control + vehicle (corn oil)p.o.; Control + CUR (1,500 mg/kg) p.o ; CaSki + vehicle (corn oil) p.o; CaSki + CUR (500 mg/kg) p.o; CaSki + CUR (1,000 mg/kg) p.o ; CaSki + CUR (1,500 mg/kg) p.o | Cervical cancer | CaSki cells injected nude mice | 500, 1000, 1500 mg/kg | / | *In vivo* | 30 days | ↓:VEGF, COX-2,  EGFR, CD31 | / | (Yoysungnoen-Chintana et al., 2014) |
|  |  |  | PTX group (20000 μmol/1); LPS group (l0mg/ml); Curcumin (50000μmol/l) | Ovarian cancer | SK0V-3/A2780 cells | 0.25, 1. 25,  2. 5 μmol/L | DMSO | *In vitro* | 12, 24, 48 h | ↓:IL-6/VEGF | TLR-4/MyD88 | (Xianlian, 2012) |
|  | Salvianolic acid B | *Salvia miltiorrhiza* Bunge (Root) [Lamiaceae] | CoCl_2_ (200μM) ; Salvianolic acid B ; CoCl_2_ (200μM) + MG132(10μM) | Cervical cancer | Siha cells | 50, 100, 150,  200 μg/ml | MEM | *In vitro* | 10, 20,  30 min, 1, 2, 3, 4,5, 6, 8, 10, 12 h | ↓: HIF-Iα, VEGF,  T-Akt, p-Akt, T-ERK1/2, p-ERK1/2 | PI3K/AKT, ERK1/2 | (Yongzhen, 2018) |

**TABLE 1-7 *(Continued)* Natural compounds antiangiogenic treatment for gynecological cancer**

| **Classifications** | **Name** | **Source**  **(authorities and family )** | **Experiments** | **Gynecological cancer** | **Animal/cell model** | **Dose range** | **Hydrotropy agent** | **Model** | **Duration** | **Molecular mechanisms** | **Signal pathways** | **Ref.** |
| --- | --- | --- | --- | --- | --- | --- | --- | --- | --- | --- | --- | --- |
| **Polyphenols** | Gallic acid | *Rhus chinensis* Mill. (Cecidium) [Anacardiaceae] | Control group; Gallic acid group | Cervical cancer | HeLa/HTB-35/  HUVEC cells | 10,15,20 µg/ml | DMSO | *In vitro* | 24 h | ↓:ADAM17, EGFR, p-Erk, pAkt | ①EGFR/  PI3K/AKT ②EGFR/MAPK/ERK | (Zhao et al., 2013) |
|  | Theaflavins-3,3'-gallate(TF3) | *Camellia sinensis* (L.) Kuntze (Leaves) [Theaceae] | NS group;  TF3 group | Ovarian cancer | OVCAR-3/ HUVEC cells | 5, 10, 15, 20, 25μM | DMSO | *In vitro* | 24 h | ↓: Akt, mTOR,  P70S6K, 4E-BP1, HIF-1α, VEGF | ①Akt/mTOR/p70S6K/  4E-BP1/HIF-lo/VEGF  ②Akt/c-Myc/Notch-1 | (Gao et al., 2016) |
|  |  |  | NS group;  TF3 group | Ovarian cancer | CAM | 25μM | DMSO | *In vivo* | 5 days | ↓:Akt, c-Myc,  Notch-1 |  |  |
|  | Cranberry proanthocyanidins（PAC-1） | *Vaccinium macrocarpon* Aiton (Fruits) [Ericaceae] | Control group;  PAC-1 group | Ovarian cancer | ①SKOV-3 cells  ②HUVEC cells | ①12.5, 25, 50, 75, 100 µg/ml  ②6.25, 12.5,  25 µg/ml | DMSO | *In vitro* | ①24 h  ②5 or 6.5 h | ↑:ROS  ↓: VEGF, AKT, p-AKT, VEGFR2 | PI3K/AKT | (Kim et al., 2012) |
|  | Green tea and (−)-epigallocatechin-3-gallate (EGCG) | *Camellia sinensis* (L.) Kuntze (Leaves)  [Theaceae] | Control group;  ET-1 group;  EGCG group | Ovarian cancer | HEY/OVCA433 cells | 10, 20, 40 μM | Distilled water | *In vitro* | 24 h | ↑:Caspase-3  ↓:Bcl-XL, cET-1, ET_A_R, VEGF, MMP2, MMP9 | ET-1/ET_A_R  autocrine signaling pathway | (Spinella et al., 2006) |
|  |  |  | Control group;  EGCG group p.o. | Ovarian cancer | HEY cells injected mice | 12.4 g/L | Distilled water | *In vivo* | 60 days | ↓:ET-1, ET_A_R, VEGF, CD31, Ki-67 |  |  |
|  |  |  | Normoxia control ( GTE and EGCG) group; Hypoxia Control (GTE and EGCG group) | Cervical cancer | HeLa cells | GTE (10, 20, 40, 80 μg/ml)  EGCG (10, 25, 50, 100 μmol/L) | Distilled water | *In vitro* | 24 h | ↓: HIF-1α, VEGF, AKT, ERK | PI3K/Akt and ERK1/2  / | (Zhang et al., 2006) |
|  |  |  | Empty plasmid group; 16 E6 group; 16 E7 group; Simulated transfection group | Cervical cancer | C-33A cells | GTE (20, 40, 80 μg/ml); EGCG (25, 50, 100 μmol/L) | Distilled water | *In vitro* | 8, 16, 24 h | ↓: HIF-1α, VEGF | / | (Tang et al., 2008) |

**TABLE 1-8 *(Continued)* Natural compounds antiangiogenic treatment for gynecological cancer**

| **Classifications** | **Name** | **Source**  **(authorities and family )** | **Experiments** | **Gynecological cancer** | **Animal/cell model** | **Dose range** | **Hydrotropy agent** | **Model** | **Duration** | **Molecular mechanisms** | **Signal pathways** | **Ref.** |
| --- | --- | --- | --- | --- | --- | --- | --- | --- | --- | --- | --- | --- |
| **Polyphenols** | Proanthocyani-dins  (BLPs) | *Myrica rubra* (Lour.) Siebold & Zucc. (Leaves) [Myricaceae] | Control group;  BLPs group | Ovarian cancer | A2780/IOSE-364/  CP70/HUVEC cells | 2.5, 5,  10 μg/mL | / | *In vitro* | 24 h | ↓:ROS, HIF-1α, VEGF, c-Myc, cyclin D1, CDK4, p-mTOR, mTOR, pp70S6K, p70S6K, p-Akt, AkT, Erk, p-4E-BP1, 4E-BP1 | Akt/mTOR/p70S6K/  4E-BP-1 | (Zhang et al., 2018) |
|  | Resveratrol (trans-3,4,5-Trihydroxystibene) | *Arachis hypogaea* L. (Fruits) [Fabaceae]  *Vitis vinifera* L. (Fruits) [Vitaceae] *Reynoutria japonica* Houtt. (Root) [Polygonaceae]  *Morus alba* L. (Fruits) [Moraceae] | Control group;  Resveratrol group | Ovarian cancer | A2780/CP70/  OVCAR-3 cells | 10, 25, 50 ,  100 μM | / | *In vitro* | 12 h | ↓: VEGF, HIF-1α, VEGF, p-AKT p-MAPK, P-p70S6K1 | PI3K/AKT/  MAPK | (Jang et al., 1997) |
| **Flavonoids** | Liquiritigenin (LQ) | *Glycyrrhiza glabra* L. [Fabaceae]  (Root) | Control group;  LQ group | Cervical cancer | HeLa cells injected  nude mice | 10, 20,  40 mg/kg | 5% sodium carboxymethycellulose solution | *In vivo* | 4 weeks | ↓: CD31, α-SMA, VEGF, PCNA | / | (Liu et al., 2012) |
|  |  |  | Control group;  LQ group | Cervical cancer | HeLa/HUVEC Cells | 25,50,75,  100 μM | DMSO | *In vitro* | 12, 24, 48 h | ↓: HIF-Iα, VEGF,  p-AKT, p-mTOR  p-p70S6K | PI3K/AKT/  mTOR-  p70S6K and ERK1/2 | (Xie et al., 2012) |
|  | Formononetin | *Astragalus mongholicus* Bunge (Root) [Fabaceae] | Control group;  DMSO group;  Formononetin group; | Cervical cancer | HeLa cells | 10, 30, 50,  100 μM | / | *In vitro* | 12, 24 h | ↓:MYC, RAS, ERK, PD-L1, STAT3, VEGF | ①RAS/ERK  ②JAK1/  STAT3 | (Wang et al., 2022) |
|  |  |  | 5-FU group (48 mg/kg); Formononetin (25, 75 mg/kg) | Cervical cancer | HeLa cells injected nude mice | 25, 75 mg/kg | Saline | *In vivo* | 30 days | ↓:PD-L1,  VEGF，MYC  p-STAT3（Tyr705）, |  |  |

**TABLE 1-9 *(Continued)* Natural compounds antiangiogenic treatment for gynecological cancer**

| **Classifications** | **Name** | **Source**  **(authorities and family )** | **Experiments** | **Gynecological cancer** | **Animal/cell model** | **Dose range** | **Hydrotropy agent** | **Model** | **Duration** | **Molecular mechanisms** | **Signal pathways** | **Ref.** |
| --- | --- | --- | --- | --- | --- | --- | --- | --- | --- | --- | --- | --- |
| **Flavonoids** | Acacetin (5,7-dihydroxy-40-methoxyflavone) | Commonly present in several plants, seeds, and flowers | Control group;  Acacetin group | Ovarian cancer | OVCAR-3/A2780 cells | 10, 20, 30 μM | DMSO | *In vitro* | 24 h | ↓: VEGF, HIF-1α, AKT, p-AKT | AKT/HIF-1α | (Liu et al., 2011) |
|  |  |  | Control group;  Acacetin group | Ovarian cancer | CAM | 10 μM | / | *In vivo* | 9 days | ↓: VEGF, HIF-1α |  |  |
|  | 7-Difluoromethyl-5,4’-dimethoxygenistein (DFMG) | *Glycine max* (L.) Merr. (Fruits) [Fabaceae] | Control group;  Solvent control group; Gen group; DFMG group; BEV group; | Cervical cancer | SiHa cells | 50 μM | / | *In vitro* | 24 h | ↓:TLR4, VEGF-A | TLR4/VEGFA | (Zhang et al., 2022) |
|  |  |  |  | Cervical cancer | CAM | 50 μM | / | *In vivo* | 24 h | ↓: The numbers of tubules and new blood vessels |  |  |
|  | Flavonoid nobiletin | *Citrus reticulata* Blanco (Fruits)  [Rutaceae] | Control group;  Flavonoid nobiletingroup | Ovarian cancer | OVCAR-3/A2780/CP70/IOSE-364 cells/ | 5,10, 20,  40, 80, 100, 160 µM | DMSO | *In vitro* | 16 h | ↓:HIF-1α, NF-κB, VEGF | PI3K/AKT | (Chen et al., 2015) |
|  |  |  | Control group;  Flavonoid nobiletin group p.o. | Ovarian cancer | CP70 cells injected athymic mice | 20 µM | / | *In vivo* | 5, 15 days | ↓:Blood vessel count |  |  |
|  | Genistein | *Glycine max* (L.) Merr. [Fabaceae] | Control group; Genistein high, middle and low dose group p.o. | Ovarian cancer | HO-8910 cells injected nude mice | 5, 25,  50 mkg/g | Sterilized deionized water | *In vivo* | 30 days | ↓:MVD, PCNA | / | (Xin, 2006) |
|  | Cardamonin | *Alpinia hainanensis* K.Schum. (Seeds) [Zingiberaceae] | Normoxia control group; CoCl_2_ control group; Normoxia rapamycin group ((0.1 µM); CoCl_2_ rapamycin group ((0.1 µM); Normoxia cardamonin group; CoCl_2_ cardamonin group | Ovarian cancer | SKOV3 cells | 1, 3, 10,  30 µM | / | *In vitro* | 24, 48 h | ↓: HIF-1α, HIF-2α, VEGF, mTOR | / | (Xue et al., 2016) |
|  |  |  | Control group (PBS); Rapamycin group (10 µM); Cardam onin group (3 µM,  30 µM, 300 µM) | Ovarian cancer | CAM | 3, 30, 300 µM | / | *In vivo* | 72 h | ↓: Blood vessels count |  |  |

**TABLE 1-10 *(Continued)* Natural compounds antiangiogenic treatment for gynecological cancer**

| **Classifications** | **Name** | **Source**  **(authorities and family )** | **Experiments** | **Gynecological cancer** | **Animal/cell model** | **Dose range** | **Hydrotropy agent** | **Model** | **Duration** | **Molecular mechanisms** | **Signal pathways** | **Ref.** |
| --- | --- | --- | --- | --- | --- | --- | --- | --- | --- | --- | --- | --- |
| **Phenylpropanoids** | Honokiol | *Magnolia officinalis* Rehder & E.H.Wilson (Root and stem bark )  [Magnoliaceae] | Control group;  Honokiol group; | Ovarian cancer | SKOV3/A2780/ COC1 cells | 5, 10, 15,  20, 25 mg/ml | / | *In vitro* | 12, 24, 36, 48, 60, 72 h | ↓:Bcl-2,Caspase-3 | / | (Li et al., 2008) |
|  |  |  | Control group (PBS 100 ml) i.p.; liposome group（0.6 mg/100 ml PBS）i.p; Honokiol group (1 mg liposome- encapsuled honokiol (40%)/100 ml PBS i.p. |  | SKOV3 cells injected nude mice | 1 mg liposome-encapsuled honokiol (40%)/100 ml PBS | / | *In vivo* | 56 days | ↓:VEGF, CD31，microvessels number |  |  |
|  | Angelol-A | *Angelica biserrata* (R.H.Shan & Yuan) C.Q.Yuan & R.H.Shan (Root) [Apiaceae] | Control group; Angelol-A group; | Cervical cancer | SiHa/HeLa/  HUVEC Cells | 40, 80, 120 μM | DMSO | *In vitro* | 24, 48 h | ↑:miR-29a-3p  ↓:MMP2, VEGFA, p-ERK1/2 | ERK/miR-29a-3p/MMP2/VEGFA | (Ying et al., 2022) |
| **Bibenzyls** | Erianin | *Dendrobium chrysotoxum* Lindl.(Stem)  [Orchidaceae] | Control group;  Erianin group;  si-PD-L-1;  si-PD-L-1+Erianin group | Cervical cancer | HeLa /HUVEC cells | 10, 30, 100 μM | / | *In vitro* | 12, 24, 48 h | ↓:TNF-α, HIF-1α, p-Akt, p-mTOR, p-p70S6K, p-4EBP1, p-eIF4,PD-L1,  VEGF, MMP-9 | ①Akt/mTOR/ p70S6K /4E-BP1  ②Raf/MEK/MAPK-ERK | (Yang et al., 2021) |
|  |  |  | Control group;  5-FU group (48 mg/kg) p.o.;  Erianin group p.o.; | Cervical cancer | HeLa cells injected nude mice | 25, 75 mg/kg | Saline | *In vivo* | 33 days | RAS, PD-L1, VEGF, HIF-1α |  |  |
| **Indoles** | Diindolylmethane (DIM) | Cruciferous vegetables | Control group;  DIM group;  cisplatin group (10 μM); DIM + cisplatin group; | Ovarian cancer | SKOV-3 cells | 50 μM | / | *In vitro* | 24 h | ↑:caspase-3, PARP  ↓: p-Tyr 705, p- Ser 727, mcl-1, survivin, VEGF, STAT-3 | STAT3/VEGF | (Kandala et al., 2011) |

**TABLE 1-11 *(Continued)* Natural compounds antiangiogenic treatment for gynecological cancer**

| **Classifications** | **Name** | **Source**  **(authorities and family )** | **Experiments** | **Gynecological cancer** | **Animal/cell model** | **Dose range** | **Hydrotropy agent** | **Model** | **Duration** | **Molecular mechanisms** | **Signal pathways** | **Ref.** |
| --- | --- | --- | --- | --- | --- | --- | --- | --- | --- | --- | --- | --- |
| **Hormones** | Melatonin (MEL) | in animals, plants, and microorganisms | Control group;  MELgroup | Ovarian cancer | SKOV3/HUVEC cells | 100,  200 μg /mL | DMEM | *In vitro* | 12, 24, 36 h | ↑:E-cadherin  ↓:MMP-9, VEGF, Vimentin | / | (Yiqing et al., 2020) |
|  |  |  | Control group (200 μL of normal saline) i.p.; MEL group i.p. | Ovarian cancer | SKOV3 cells injected nude mice | 25 mg /kg | / | *In vivo* | 3 weeks | CD31 |  |  |
| **Macrolides** | Rapamycin | Soil in the Wai Atari region of Rapa Nui (Easter Island) | NS group, DDP group (10 μmol/L);  Rapamycin group  (40 nmol/L );  Combination group | Ovarian cancer | SKOV-3 cells | 40 nmol/L | DMEM/F12 | *In vitro* | 24 h | ↓:VEGFA, ATP,VEGFB,  VEGFC | / | (Zhonghua, 2015) |
